# *Drosophila *EGFR pathway coordinates stem cell proliferation and gut remodeling following infection

**DOI:** 10.1186/1741-7007-8-152

**Published:** 2010-12-22

**Authors:** Nicolas Buchon, Nichole A Broderick, Takayuki Kuraishi, Bruno Lemaitre

**Affiliations:** 1 Global Health Institute, École Polytechnique Fédérale de Lausanne, Lausanne, Switzerland

## Abstract

**Background:**

Gut homeostasis is central to whole organism health, and its disruption is associated with a broad range of pathologies. Following damage, complex physiological events are required in the gut to maintain proper homeostasis. Previously, we demonstrated that ingestion of a nonlethal pathogen, *Erwinia carotovora carotovora 15*, induces a massive increase in stem cell proliferation in the gut of *Drosophila*. However, the precise cellular events that occur following infection have not been quantitatively described, nor do we understand the interaction between multiple pathways that have been implicated in epithelium renewal.

**Results:**

To understand the process of infection and epithelium renewal in more detail, we performed a quantitative analysis of several cellular and morphological characteristics of the gut. We observed that the gut of adult *Drosophila *undergoes a dynamic remodeling in response to bacterial infection. This remodeling coordinates the synthesis of new enterocytes, their proper morphogenesis and the elimination of damaged cells through delamination and anoikis. We demonstrate that one signaling pathway, the epidermal growth factor receptor (EGFR) pathway, is key to controlling each of these steps through distinct functions in intestinal stem cells and enterocytes. The EGFR pathway is activated by the EGF ligands, Spitz, Keren and Vein, the latter being induced in the surrounding visceral muscles in part under the control of the Janus kinase/signal transducer and activator of transcription (JAK/STAT) pathway. Additionally, the EGFR pathway synergizes with the JAK/STAT pathway in stem cells to promote their proliferation. Finally, we show that the EGFR pathway contributes to gut morphogenesis through its activity in enterocytes and is required to properly coordinate the delamination and anoikis of damaged cells. This function of the EGFR pathway in enterocytes is key to maintaining homeostasis, as flies lacking EGFR are highly susceptible to infection.

**Conclusions:**

This study demonstrates that restoration of normal gut morphology following bacterial infection is a more complex phenomenon than previously described. Maintenance of gut homeostasis requires the coordination of stem cell proliferation and differentiation, with the incorporation and morphogenesis of new cells and the expulsion of damaged enterocytes. We show that one signaling pathway, the EGFR pathway, is central to all these stages, and its activation at multiple steps could synchronize the complex cellular events leading to gut repair and homeostasis.

## Background

An important function of epithelial surfaces is to maintain the barrier between an organism's internal and external environments. This is especially true for the gut epithelium because of the magnitude of its surface and exposure to both ingested material and the indigenous microbiota [1,2]. Gut integrity is maintained in large part through epithelial renewal, which is sustained by the proper activation and differentiation of stem cells embedded in the gut [1]. In mammals, the intestinal epithelium displays one of the most rapid turnover rates of any tissue. Stem cells, located in the basal crypts, proliferate continuously to completely turn over the gut every 3 to 4 days [3]. Similar to mammals, the *Drosophila *adult midgut is sustained by intestinal stem cells (ISCs), which self-renew and produce a population of nondividing, undifferentiated ISC daughters, termed *enteroblasts *[4,5]. Some of these progenitors remain in a transient state in the gut, while the majority differentiate into the two principal cell types of the intestinal epithelium: absorptive enterocytes and secretory enteroendocrine cells. The turnover of enterocytes is continuous, and it is thought that the entire *Drosophila *gut epithelium is renewed in 7 to 10 days [5]. In addition to this function in basal maintenance, epithelial renewal is also critical in the host response to acute damage to the gut.

In this line, several reports have demonstrated that, in *Drosophila*, ingestion of cytotoxic compounds or damage by enteric pathogens increases epithelial renewal through ISC proliferation [6-11]. We and others further demonstrated that the Janus kinase/signal transducer and activator of transcription (JAK/STAT) pathway is required for bacteria-induced epithelium renewal [7,9,10]. These studies showed that stressed and/or damaged enterocytes produce a secreted ligand, Upd3, which activates the JAK/STAT pathway in ISCs to promote both their division and their differentiation, establishing a homeostatic regulatory loop. Interestingly, flies unable to renew their epithelium succumb to infection, demonstrating that epithelium renewal is an essential component of *Drosophila *defense against oral bacterial infection [7,9,10]. These studies also identified natural stimuli that provoke ISC activation, providing a powerful model system to study epithelium renewal and its genetic control.

Despite these studies, it remains unclear how ISC activation and epithelium renewal are globally coordinated. Gut repair upon bacterial infection not only involves the proliferation of ISCs but also necessitates the elimination of damaged cells and integration of new cells into the epithelium, two processes that have not received attention. Moreover, genome-wide profiling of the gut response to damage caused by infection indicates that other pathways are induced [8], suggesting that additional pathways could contribute to the regulation of epithelium renewal.

In the present work, we have measured the morphological changes that occur in the gut of *Drosophila *in response to ingestion of a nonlethal pathogenic bacterium, *Erwinia carotovora carotovora 15 *(*Ecc15*). We show that infection induces a dramatic remodeling of the gut, which is required to repair the loss of nearly half its cells. This repair occurs initially through the immediate differentiation of enteroblasts, a pool of undifferentiated progenitors, and is completed through stem cell proliferation and differentiation. Our study demonstrates the complexity of epithelium renewal in response to infection, as it encompasses three different processes: (1) the proliferation and differentiation of ISCs, (2) the proper morphogenesis of new enterocytes, and (3) the delamination and anoikis of damaged enterocytes. We further demonstrate that one signaling pathway, the epidermal growth factor receptor (EGFR) pathway, is key to controlling these three cellular and morphogenetic events, therefore ensuring gut homeostasis following infection.

## Results

### Infection with *Ecc15 *induces a major remodeling of the gut

**Figure 1:**
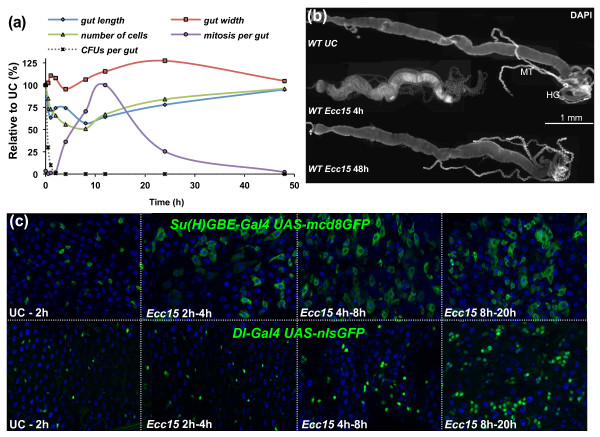
**Ingestion of *Erwinia carotovora carotovora 15 *(*Ecc15*) induces dramatic morphological changes to the gut of *Drosophila***. **(a) **Quantitative measurements of the gut at different time points after infection reveal that *Ecc15 *induces a dramatic remodeling of the gut. The midgut lengths and widths, the total number of cells and the number of mitotic stem cells (phosphohistone H3 (PH3)-positive cells) along the midgut are shown. Measurements of colony-forming units (CFUs) per gut of *Ecc15 *at corresponding time points are also shown. **(b) **Representative images of guts dissected from wild-type (WT), unchallenged (UC) or *Ecc15*-infected flies stained with 4',6-diamidino-2-phenylindole (DAPI) and observed by light microscopy at ×10 original magnification. Gut length is decreased at 4 hours after infection, but returns to unchallenged levels by 48 hours. MT, Malpighian tubules; HG, hindgut. **(c) **Expression of green fluorescent protein (GFP) under the control of enteroblast (*Su(H)GBE-Gal4*; *UAS-mcd8GFP*) or intestinal stem cell (ISC) (*delta-Gal4*; *UAS-nlsGFP*) specific reporter genes was monitored following infection with *Ecc15*. Soon after infection (> 2 hours), expansion of *Su(H)GBE-Gal4*; *UAS-mcd8GFP *GFP signal was observed along the gut, reflecting the rapid differentiation of enteroblasts into larger enterocytes. The expansion of *delta-Gal4*; *UAS-nlsGFP *was observed only after 4 hours, indicative of ISC proliferation.

To understand the precise cellular events that occur in the gut following infection, we performed a quantitative analysis of gut remodeling following the ingestion of *Ecc15*. We measured gut length and width, the number of mitotic events (as indicated by phosphohistone H3 (PH3)-positive small nucleated cells), and the relative numbers of precursor cells (ISCs and enteroblasts, expressing the *escargot *marker), young nonpolyploid enterocytes, and mature polyploid enterocytes. Total numbers of intestinal cells decreased as early as 30 minutes postinfection and reached their lowest point by 8 hours, with a loss of approximately half of the cells (Figure 1a and Additional files 1 and 2). This dramatic loss of cells was correlated with a striking shortening of the gut (reduced by 40%) and an increase in gut width (Figures 1a and 1b and Additional file 1B). Interestingly, in the 2-hour to 4-hour time period, a short and transient elongation of the gut was observed, despite the continued decrease in cell numbers (Figure 1a and Additional file 1B). This transient extension began 1 to 2 hours postinfection and was correlated with an increase in the proportion of mature enterocytes concomitant with a decrease in precursor cells (Additional files 1D and 2B). In addition, it was not caused by stem cell proliferation, since the wave of mitosis began 4 hours postinfection, after this transient elongation was already achieved (Additional file 1). The role of the precursor population in the transient extension was supported by the increase in the size of cells expressing an enteroblast-specific marker (*Su(H)GBE-Gal4 UAS-mcd8GFP*) due to their differentiation to large enterocytes (Figure 1c). In contrast, no change in an ISC-specific marker (*delta-Gal4 UAS-nlsGFP*) was observed at early time points (1 to 4 hours; Figure 1c). This indicates that, upon infection, the first phase of gut repair results from the immediate mobilization of a pool of preexisting enteroblasts and their differentiation into enterocytes.

At 8 hours postinfection, the total number of gut cells began increasing as a consequence of stem cell proliferation, which peaked 12 hours postinfection (Figure 1a and Additional files 1C and 2A). Furthermore, we observed an expansion of the green fluorescent protein (GFP) signal arising from an ISC-specific marker (*delta-Gal4 UAS-nlsGFP*) (Figure 1c), which correlates with the increase in mitotic index in these guts. ISC proliferation and their subsequent differentiation into enterocytes were sufficient to restore the morphology of the gut to preinfection dimensions by 48 hours postinfection (Figures 1a and 1b). However, the proportions between different cell populations in the gut remained different from unchallenged conditions, but were restored by 120 hours postinfection (Additional files 1D and 2B). These results indicate that, following infection with *Ecc15*, epithelium repair occurs as a biphasic response, comprised of an immediate differentiation of preformed progenitor cells followed by epithelial replenishment through ISC proliferation and differentiation.

**Figure 2:**
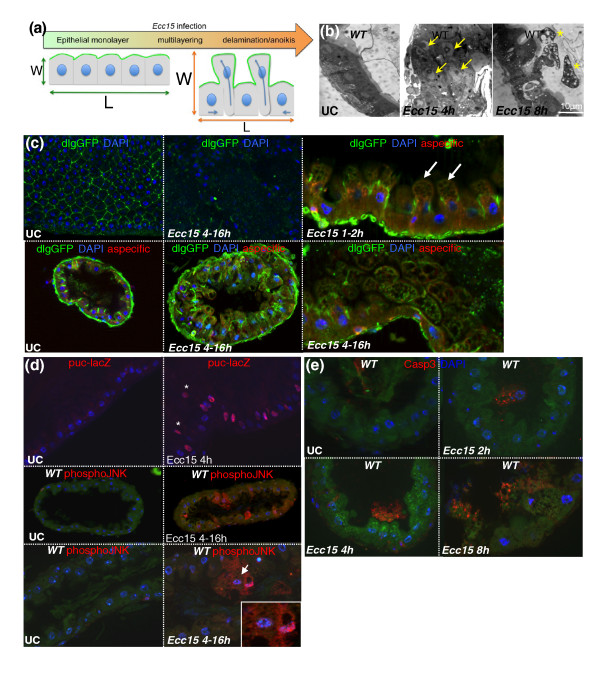
**Ingestion of *Ecc15 *results in the delamination of enterocytes and anoikis**. **(a) **A scheme describing the mechanism by which delamination of enterocytes could account for the shortening and widening of the gut. W, width; L, length. **(b) **Infection results in an intense multilayering (arrows) and the blebbing (stars) and delamination of enterocytes following ingestion of *Ecc15*. Representative images of UC and *Ecc15 *infected (*t *= 4 and 8 hours) guts are shown. Histological sections of the anterior midgut region were analyzed by light microscopy at ×63 original magnification. (c) Localization of the septate junction marker Discs large (*dlgGFP*) reveals a regular pattern in UC WT flies. Cross section of the gut shows that Dlg is located apically between enterocytes. Infection with *Ecc15 *disrupts Dlg localization in the gut. Cell blebbing (white arrows) occurs apically to the Dlg compartment. **(d) **Immunostaining of guts expressing the c-Jun N-terminal kinase (JNK)-responsive reporter gene *puc-lacZ *revealed that ingestion of *Ecc15 *results in the activation of the JNK pathway in most enterocytes and all delaminating cells. Additionally, immunostaining of sections of guts from WT flies with antibodies directed against the phospho-form of JNK (red) reveals continued JNK activation in delaminating enterocytes (4 to 16 hours postinfection with *Ecc15*). Fragmented nuclei were observed in some of these JNK-positive cells (inset). **(e) **Immunostaining of histological sections of WT flies with antibodies directed against the cleaved form of caspase 3 shows that cells undergo apoptosis only after they have detached from the epithelium. This signal was detected during the duration of the infectious process in cells at varying stages of delamination and anoikis.

The correlation between the amplitude of cell loss and reduction in gut length led us to hypothesize that the gut widening and shortening was mainly caused by enterocyte delamination, as depicted in Figure 2a. In agreement, we observed that ingestion of *Ecc15 *led to the multilayering of epithelial cells, followed by their sloughing and accumulation in the space between the epithelium and the peritrophic matrix (a chitinous barrier lining the gut of insects) (Figure 2b and Additional File 3). In addition, infection led to the detachment of GFP-expressing enterocytes from the gut (Additional File 4A), and the sloughing of cells into the lumen was correlated with the appearance of a characteristic yellow autofluorescence along the gut (Additional file 4C). Closer examination of guts at 1 to 2 hours postinfection revealed the blebbing of cells (Figure 2c, white arrow), a process commonly observed during cell death [12,13]. This cell blebbing occurred through the extension of the apical domain of cells, as blebs were located more apically than the septate junction marker Discs large (dlg; Figure 2c, right top). At later time points (4 to 16 hours), infection resulted in the loss of dlg staining, indicating a loss of cell polarity and the disruption of septate junctions (Figure 2c). We observed a large number of cells detaching from the basal epithelium layer and shed into the lumen. To further characterize this process, we examined c-Jun N-terminal kinase (JNK) pathway activity, as JNK is a stress-responsive pathway induced in both ISCs and enterocytes in the gut upon infection [8]. We detected JNK activation in these delaminating cells, as measured by *puc-LacZ *expression and phospho-JNK detection (Figure 2d). This indicates that, in addition to the early JNK activation (30 minutes postinfection) that has been reported in response to infection [7], there is prolonged JNK activation in a subset of enterocytes that eventually delaminate. In addition, these cells had an abnormal morphology and fragmented nuclei and contained multiple large vacuoles, suggesting they were engaged in a cell death process (Figures 2b and 2d (inset) and Additional file 3B). In support of this hypothesis, these detached cells were positive for caspase 3 activity, indicative of apoptosis (Figure 2e). Importantly, cell destruction by apoptosis (nuclear fragmentation or absence of nucleus and punctate caspase 3 signal) was apparent only in cells detached from the epithelium (see Figure 2e for quantification). Histological sections revealed that the vacuoles in detached cells frequently contained double membranes (Additional file 5A), suggesting they were autophagic. The induction of autophagy was confirmed by the increased detection of punctae enriched in markers for autophagy (lysosome-associated membrane protein (Lamp), light chain associated protein 3 (LC3) and *autophagy related gene 8 *(ATG8) in delaminating cells (Additional file 5B). These results are in agreement with a previous study in *Drosophila *demonstrating that programmed cell death in the larval gut is associated with increased caspase activity and autophagy [14]. Together these data support the hypothesis that infection with *Ecc15 *leads to the delamination of damaged enterocytes and their subsequent death in the lumen through anoikis, a specific process of apoptosis induced by the loss of cell attachment. This process of cell blebbing, delamination and anoikis occurs as a sequence of events for a given cell, but continues over the duration of the infection as the gut returns to its preinfection state.

Altogether, our quantitative analysis reveals the profound plasticity of the *Drosophila *gut, which maintains its integrity and barrier function despite the loss of half of its cells. This integrity is maintained through the coordinated removal of dead cells by anoikis and their replacement through a biphasic repair response.

### The EGFR/mitogen-activated protein kinase pathway is required cell-autonomously to promote ISC proliferation induced by infection

**Figure 3:**
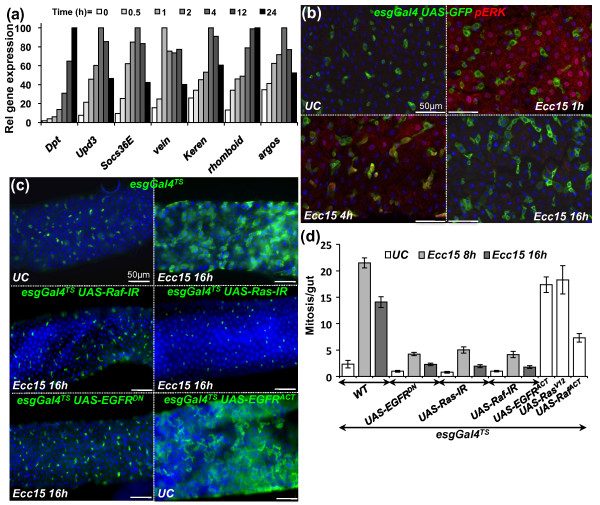
**The epidermal growth factor receptor (EGFR) pathway is induced in the gut and is required for ISC proliferation triggered by infection**. **(a) **Reverse transcriptase-quantitative polymerase chain reaction (RT-qPCR) analysis of gut extracts shows that genes encoding components of the Imd (*Diptericin *(*Dpt*)), Janus kinase/signal transducer and activator of transcription (JAK/STAT) (*upd3*, *Socs36E*) and EGFR (*vein*, *Keren*, *rhomboid*, *argos*) pathways are induced upon oral ingestion with *Ecc15*. Values were normalized to *RpL32 *and set relative to their own maximum induction levels. **(b) **Immunostaining of guts of *esgGal4 UAS-GFP *flies with antibodies directed against the phospho-form of the extracellular signal-regulated kinase (ERK) kinase (red) and GFP (green) reveals that the ERK kinase was activated in enterocytes 1 hour postinfection and in both progenitor cells (green) and enterocytes at 4 hours. At 16 hours, no phospho-ERK signal was detected. **(c) **Ingestion of *Ecc15 *induces a marked increase in the number of *esgGal4^TS^*, *UAS-GFP*-positive cells (indicative of epithelium renewal), which was not observed when double-stranded RNA (dsRNA or RNAi) or dominant-negative forms of members of the EGFR pathway (*UAS-EGFR^DN^*, *UAS-Ras-IR *or *UAS-Raf-IR*) were expressed in ISCs. Overexpression of an active form of EGFR (*UAS-EGFR^ACT^*) in ISCs is sufficient to induce a high level of epithelium renewal in the absence of infection. **(d) **Quantification of PH3-positive cells per midgut shows an increase in the number of mitotic cells upon *Ecc15 *infection in WT flies, but not in flies with reduced EGFR activity in ISCs (*UAS-EGFR^DN^*, *UAS-Ras-IR *or *UAS-Raf-IR*; *P *< 0.05). Overexpression of a constitutively activated form of EGFR in ISCs increases the mitotic index (*esgGal4^TS^*, *UAS-EGFR^ACT^*, *UAS-Ras^V12 ^*or *UAS-Raf^ACT^*; *P *< 0.05). Mean values of five experiments (*N *= 10 to 20 guts each) ± SE are shown. Analysis of variance (ANOVA) *F *= 58.64. *df *= 14. *P *< 0.0001.

The profound gut remodeling observed upon bacterial infection suggests the existence of regulatory mechanisms that coordinate the elimination of cells through delamination and the integration of new cells into the epithelium, all while maintaining gut barrier function and integrity. The EGFR signaling cascade is one of the cardinal pathways regulating cell remodeling during development in a broad range of multicellular organisms [15]. In *Drosophila*, EGFR is the sole receptor of the pathway, and the downstream cascade is the canonical Ras/Raf/MAPK kinase kinase (MEKK)/extracellular signal-regulated kinase (ERK) pathway (in *Drosophila: Ras85D/pole hole/MEKK/rolled*, respectively) [15]. Several members of the EGFR pathway are induced at the transcriptional level in the gut upon *Ecc15 *infection [8] (Figure 3a). This includes the transcription factor Pointed, the EGFR ligands Vein and Keren, and the protease Rhomboid, which is known to positively maturate and activate EGFR ligands, as well as the negative regulator Argos (Figure 3a). To further confirm the activation of the EGFR/mitogen-activated protein kinase (MAPK) pathway upon infection, we quantified the levels of phosphorylated (active) forms of the terminal MAPK, ERK, in the gut by immunolocalization with an antibody specific to its phospho-form. Signals of phospho-ERK were detected in enterocytes 1 hour postinfection (Figure 3b). At 4 hours postinfection, ERK activity was observed in some *escargot-GFP*-positive precursor cells in addition to some enterocytes. ERK activity returned to unchallenged levels at 16 hours postinfection. The dynamics of ERK activity indicate that ingestion of *Ecc15 *activates the EGFR pathway in both enterocytes and precursor cells.

The activation of the EGFR pathway in progenitor cells led us to examine the role of this pathway in controlling ISC activity upon bacterial infection. To examine this, we expressed double-stranded RNA (dsRNA or RNAi) or dominant-negative constructs targeting EGFR pathway components in ISCs using an *esgGal4^TS^*, which expresses *Gal4 *under thermosensitive conditions (see Materials and Methods). Inactivation of the EGFR/MAPK pathway with *UAS-EGFR^DN^*, *UAS-Ras-IR *and *UAS-Raf-IR *in ISCs did not significantly affect the number of ISCs in the gut (Figure 3c and Additional file 6A) or the mitotic index in unchallenged conditions (Figure 3d). However, in sharp contrast to wild-type flies, *Ecc15 *infection did not increase the number of mitotic cells (PH3-positive; Figure 3d) or change the pattern and distribution of *escargot-GFP*-positive cells (Figure 3c) in the gut of flies with reduced EGFR signaling in ISCs, indicating a lack of induction of epithelium renewal. Similarly, ectopic expression of Argos or the phosphatase MAPK phosphatase 3 (MKP3) (two negative regulators of the EGFR pathway), as well as RNAi against the transcription factor Pointed, also blocked ISC proliferation induced by *Ecc15 *(Additional file 6A), indicating that a canonical EGFR/MAPK pathway acts in ISCs to promote proliferation. In agreement with these observations, flies with reduced levels of EGFR pathway activity in ISCs exhibited a higher mortality to *Ecc15 *infection because of a failure in gut repair (Additional file 7). Conversely, overexpression of an activated form of the EGFR receptor (*UAS-EGFR^ACT^*) in ISCs was sufficient to induce proliferation along the gut in the absence of infection (Figures 3c and 3d), although the resulting cells are abnormally shaped and do not appear as fully differentiated enterocytes (Additional file 6B). Altogether, these results indicate that the EGFR pathway is both required and sufficient in ISCs to promote their proliferation in response to infection.

### Multiple EGFR ligands are involved in ISC proliferation

A key mechanism of EGFR pathway activation is through binding of EGFR ligands (EGFs) [15]. *Drosophila *has four EGFs: Spitz, Keren, Gurken and Vein. Vein is produced as a secreted protein that does not require further processing, while the other three ligands require maturation by the protease Rhomboid. *Vein*, *Keren *and *rhomboid *are transcriptionally induced in the gut upon infection with *Ecc15 *[8] (Figure 3a), while *spitz *but not *gurken *is expressed in the gut (data not shown), indicating that three of the four ligands could potentially activate the EGFR pathway in the adult gut.

**Figure 4:**
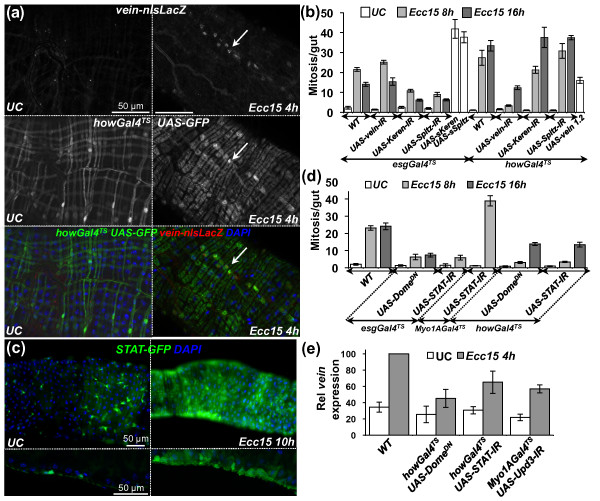
**The JAK/STAT pathway is required for *vein *expression in the visceral muscles upon infection with *Ecc15***. **(a) **Immunostaining against lacZ and GFP of guts derived from *vein-lacZ*; *howGal4^TS ^UAS-GFP *flies reveals than *vein *(nuclear signal) is induced upon infection in the circular visceral muscles. **(b) **RNAi silencing of *vein *in visceral muscles and reduction of *Keren *and *Spitz *in precursor cells blocked infection-induced proliferation (*P *< 0.05). Conversely, ectopic expression of the three EGFs (*UAS-sKeren*, *UAS-sSpitz *and *UAS-vein^1.2^*) was sufficient to trigger proliferation (*P *< 0.05). Mean values from five experiments (*N *= 10-20 guts each) ± SE are shown. ANOVA *F *= 62.96. *df *= 26. *P *< 0.0001. **(c) **Infection induced the JAK/STAT reporter gene, *STAT-GFP*, in visceral muscles in addition to ISCs. Representative images of UC and *Ecc15*-infected guts are shown. Guts of *STAT-GFP *flies were stained with DAPI and examined by fluorescence microscopy at ×20 original magnification. The microscopic focus was set to the external layer of the gut (top). Transverse sections show that expression of *STAT-GFP *is localized to the circular visceral muscle and progenitor cells (bottom). **(d) **The JAK/STAT pathway is required in both ISCs and visceral muscles to promote ISC proliferation. RNAi directed against *STAT92E *or expression of a dominant-negative form of *Domeless *in ISCs (*esgGal4^TS^*) or muscles (*howGal4^TS^*), but not enterocytes (*Myo1AGal4^TS^*) strongly reduced the number of mitotic ISCs in the guts of flies infected with *Ecc15 *(*t *= 16 hours; *P *< 0.05). Mean values from five experiments (*N *= 10-20 guts each) ± SE are shown. ANOVA *F *= 50.74. *df *= 15. *P *< 0.0001. **(e) **The induction of *vein *upon *Ecc15 *infection was reduced in flies with reduced JAK/STAT signaling in visceral muscles (*P *< 0.05). Tissue-specific silencing of *upd3 *in enterocytes also reduced *vein *expression (*P *< 0.05). RT-qPCR analysis of gut extracts from UC and *Ecc15*-infected flies (*t *= 8 hours). Levels of *vein *expression were normalized to *RpL32*. Mean values from four experiments (*N *= 20 guts each) ± SE are shown. ANOVA *F *= 14.83. *df *= 7. *P *< 0.0001.

We next examined where these EGFs are produced in the adult midgut. Immunostaining against β-galactosidase of *vein-lacZ *flies revealed that *vein *is induced in the inner layer of circular visceral muscles surrounding the midgut (Figure 4a). This is consistent with a previous study showing that in larvae *vein *is produced in visceral muscles and promotes the proliferation of adult midgut precursor cells [16]. Use of *spitz-Gal4 UAS-GFP *flies revealed that *spitz *was expressed in precursor cells (Additional file 8A). The expression pattern of *Keren *was not directly assessed because of the lack of a reporter gene. Nevertheless, we used an *in vivo *RNAi approach to deplete the three EGFs individually in three different populations of gut cells: visceral muscles (*howGal4^TS^*), precursor cells (*esgGal4^TS^*) or enterocytes (*Myo1AGal4^TS^*). Interestingly, reduced epithelium renewal and ISC proliferation were observed only with knockdown of the expression of *vein *in visceral muscles and *Keren *and *spitz *in progenitor cells (Figure 4b, Additional File 6A and 8B and data not shown). Nevertheless, none of the RNAi targeting a single EGF fully blocked ISC proliferation induced by infection. Conversely, expression of *vein *in the visceral muscles slightly stimulated ISC proliferation, and expression of the constitutively active forms of Spitz and Keren in precursor cells induced high levels of proliferation (Figure 4b). Altogether, these results suggest that the three EGFs participate in the activation of the EGFR pathway in ISCs to promote their proliferation; one of them, *vein*, is expressed in the inner visceral muscles, while *Keren *and *spitz *are expressed within the epithelium.

### Expression of *vein *in visceral muscles is mediated by the JAK/STAT pathway

The JAK/STAT pathway is activated in ISCs through the release of Upd3 by damaged enterocytes and controls both proliferation and differentiation of ISCs upon infection [7,9,10]. Use of a *STAT-GFP *reporter transgene revealed that the JAK/STAT pathway is activated not only in ISCs, as previously described [7,10,17-19], but also in some visceral muscles in response to infection (Figure 4c and Additional file 9). Additionally, specific knockdown of the JAK/STAT pathway using a dominant-negative form of the receptor Domeless or a *STAT-RNAi *in the visceral muscles reduced ISC proliferation (Figure 4d), uncovering a new role of the JAK/STAT pathway in this tissue. We hypothesize that the production of Upd3 by enterocytes could activate the JAK/STAT pathway in visceral muscles, which would then indirectly regulate ISC proliferation through the production of the EGF Vein. In agreement with this hypothesis, depletion of JAK/STAT activity in visceral muscles or reduction of *upd3 *in enterocytes partially decreased the level of *vein *expression (Figure 4e). These results indicate that, in addition to its role in progenitor cells, the JAK/STAT pathway indirectly contributes to ISC proliferation through the transcriptional activation of the EGF *vein *in visceral muscles.

### The JAK/STAT and EGFR pathways synergize in ISCs to promote cell proliferation

**Figure 5:**
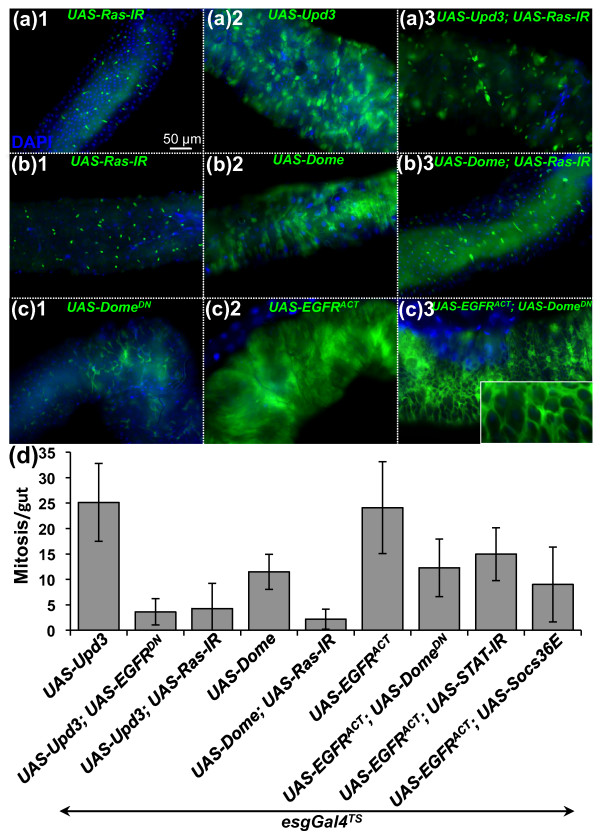
**The EGFR and JAK/STAT pathways synergize to promote ISC proliferation**. Expression of the *esgGal4^TS^*, *UAS-GFP *reporter gene **(a-c) **and quantification of PH3-positive cells per midgut **(d) **were monitored in UC flies. Overexpression of *upd3 *(A2) or *domeless *(B2) in ISCs induced higher levels of epithelium renewal in UC flies, which was reduced by depletion of EGFR or Ras activity (A3 and B3). Conversely, the high levels of epithelium renewal induced by overexpressing an activated form of the EGFR (C2) in ISCs was slightly reduced by coexpression of a negative regulator of the JAK/STAT pathway (*UAS-Socs36E*), a dominant-negative form of Domeless (*UAS-Dome^DN^*) or a *UAS-STAT-IR *construct (C3 and D). Ectopic expression of the EGFR in ISCs expressing a dominant-negative form of Domeless did not rescue the differentiation defect caused by the lack of JAK/STAT activity, but instead aggravated the expansion of undifferentiated *escargot-GFP *precursor cells (C3 and inset).

The JAK/STAT and EGFR pathways are both activated in precursor cells following ingestion of *Ecc15*. We performed an epistatic analysis to determine the relationship between these two pathways. Ectopic expression of the Upd3 cytokine in ISCs strongly increased both the mitotic index and epithelium renewal to levels similar to or higher than that observed in infected guts [7,10] (Figures 5a2 and 5d). Reduction of the EGFR/MAPK pathway in ISCs using either *UAS-Ras-IR *or *UAS-EGFR^DN ^*blocked the epithelium renewal induced by Upd3 (Figures 5a3 and 5d). Similarly, expression of either *UAS-Ras-IR *or *UAS-EGFR^DN ^*in ISCs suppressed ISC proliferation induced upon overexpression of the JAK/STAT receptor Domeless in ISCs (*UAS-Dome*) (Figures 5b2, 5b3 and 5d). These experiments indicate that the EGFR pathway acts downstream from or parallel to JAK/STAT signaling in the control of compensatory ISC proliferation. Conversely, the mitotic index in the gut of flies overexpressing a constitutively active form of EGFR in ISCs was decreased but not abolished when JAK/STAT pathway activity was reduced in ISCs (using *UAS-Dome^DN^*, *UAS-STAT-IR *or *UAS-Socs36E*; Figure 5d). We conclude that the JAK/STAT pathway is required for the full activation of ISCs by the EGFR pathway. In addition to its role in ISC proliferation, the JAK/STAT pathway is also required for the differentiation of progenitors into mature enterocytes [7,10,17-19]. As a consequence, flies with a reduced JAK/STAT pathway in ISCs show tumorlike accumulation of small, undifferentiated *escargot-GFP*-positive cells [7,10,17,18]. Overexpression of the activated form of the EGFR pathway in ISCs did not restore the differentiation defect provoked by a reduction in JAK/STAT signaling, but instead aggravated the spreading of the tumorlike progenitors (Figure 5c3). Our epistatic analysis was confirmed using the *esg^TS^-Flip-Out *system, a method of selectively activating a ubiquitous Gal4 driver in all progenitor cells and their subsequent progeny [10] (Additional file 10). These data indicate that activation of both the EGFR and JAK/STAT pathways simultaneously is required for full proliferation of ISCs. In addition, the JAK/STAT pathway has a unique function in the differentiation of progenitors, which is not rescued by ectopic activation of the EGFR pathway.

### EGFR pathway is required in enterocytes for proper adult gut morphogenesis

**Figure 6:**
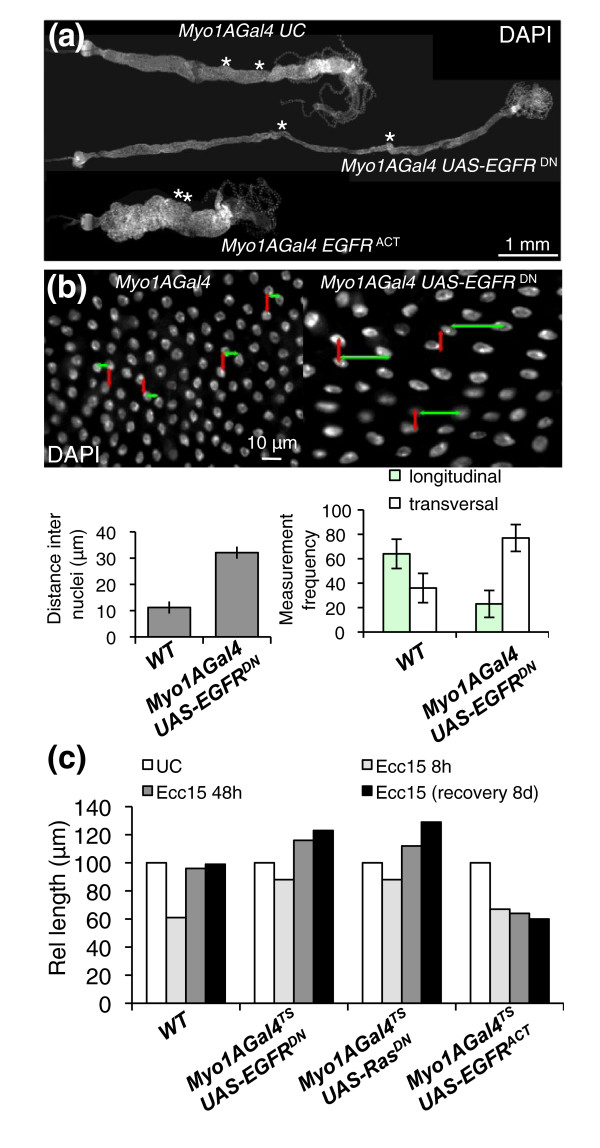
**The EGFR pathway is required in enterocytes for proper gut morphogenesis**. **(a) **Midguts with enterocytes depleted of the EGFR pathway (*Myo1AGal4*, *UAS-EGFR^DN^*) are longer and thinner than WT guts. Conversely, guts from flies expressing a constitutive form of EGFR are shorter and wider. The effect of EGFR is most pronounced in the copper cell region (borders of which are indicated with asterisks). Representative images were taken of dissected guts from 3-day-old to 4-day-old flies stained with DAPI. Guts from flies expressing a constitutive form of EGFR are approximately half the length of WT UC flies. **(b) **Nuclear staining of guts with EGFR-deficient enterocytes reveals a decrease in cell density and flattening of nuclei as compared to WT guts. Quantification of the mean distance between the nearest adjacent nuclei in WT and EGFR-depleted guts. Measures were taken in the region around the copper cells (middle midgut), where the effect of EGFR is most pronounced (Additional file 11C). Additionally, the orientation of the distance vector between the two nearest nuclei switches from longitudinal to transversal, indicating a change in epithelial geometry (see model, Additional file 11D). **(c) **Relative length of WT guts, guts with enterocytes depleted for the EGFR pathway or guts with activated EGFR in enterocytes. Constructs were placed under the control of a thermosensitive enterocyte driver (*Myo1AGal4^TS^*). Adult flies were switched from 18°C (Gal4 nonfunctional) to 29°C (Gal4 functional) 3 days before infection or before initial measurements were taken. Guts depleted for the EGFR pathway (*UAS-EGFR^DN ^*or *UAS-Ras^DN^*) in enterocytes shrink less upon infection with *Ecc15*. After a recovery phase of 2 or 8 days, the guts are 25% longer than their WT counterparts. Conversely, guts of flies with ectopic activation of EGFR (*UAS-EGFR^ACT^*) in enterocytes do not elongate in response to infection and are 40% shorter than WT guts.

The EGFR pathway is also activated in enterocytes following bacterial infection (Figure 3b). To analyze the role of EGFR pathway in enterocytes, we reduced EGFR activity in enterocytes using the driver *Myo1AGal4 *in combination with *UAS-EGFR^DN^*. Strikingly, guts from flies with reduced EGFR activity in enterocytes were almost double the length and half the width of wild-type flies (Figure 6a and Additional file 11A). However, the total number of enterocytes remained constant (data not shown). Further observations indicated that this alteration in gut length was correlated with changes in the shape of enterocytes. More specifically, enterocytes with reduced EGFR activity appeared flattened and to have lost the extension of their apical domain, as revealed by actin and membrane-associated GFP staining (Additional file 11B). In addition, the distance between nuclei of neighboring enterocytes (indicative of epithelial density) increased twofold in flies with reduced EGFR activity and was altered in its axis (Figure 6b). This effect on cell shape was more pronounced in certain gut segments, particularly around the copper cells region (Figure 6a, asterisks, and Additional file 11), which is normally composed of a highly polarized columnar type of epithelium and is folded within the abdomen. As a consequence, guts of flies with reduced EGFR in enterocytes were long and thin in this region and lacked their characteristic folding (Figure 6a). Importantly, flies lacking EGFR in enterocytes succumbed within 48 hours after ingesting *Ecc15 *(Additional file 12A). This increased susceptibility was associated with a rupture in gut integrity, as indicated by the presence of bacteria in the hemolymph (Additional file 12B). Increased gut length was also observed in flies expressing a *UAS-Ras^DN ^*construct in enterocytes, suggesting the role of both EGFR and downstream components (Additional file 11A). Conversely, ectopic activation of EGFR in enterocytes resulted in adult flies with guts two times shorter and three times wider than wild-type fly guts (Figure 6a).

The above experiments point to a role of EGFR in enterocyte morphogenesis. Nevertheless, they rely on the use of the *Myo1AGal4 *driver that continuously expresses Gal4 along the entire development. As a consequence, we could not conclude whether the effect of the EGFR pathway occurred in mature enterocytes, in enterocytes undergoing maturation during epithelium renewal or earlier during the development of the gut. Therefore, we examined the impact of EGFR using the *Myo1AGal4 *thermosensitive (*Myo1AGal4^TS^*) driver, which enabled us to activate or downregulate the EGFR pathway specifically in adult enterocytes to avoid developmental effects. Guts of *Myo1AGal4^TS^*; *UAS-EGFR^DN ^*flies shifted to the restrictive temperature for 4 to 5 days are initially of normal size, indicating that the effect of EGFR on the gut was not due to an immediate alteration in the shape of mature enterocytes (Figure 6c). However, we observed a significant increase in gut length when flies were incubated for 3 weeks at the restrictive temperature (Additional file 11E), a time frame in which there is significant renewal of the gut during normal aging. This suggests that the requirement of the EGFR pathway for proper enterocyte shape occurs during their maturation. To confirm this observation, we analyzed the gut length of *Myo1AGal4^TS^*; *UAS-EGFR^DN ^*flies orally infected with *Ecc15*, a challenge that considerably accelerates cell renewal in the gut. Figure 6c shows that 48 hours postinfection, "regenerated" guts from flies with reduced EGFR signaling in enterocytes were 20% longer than in wild-type flies. Conversely, guts of flies overexpressing an activated form of EGFR were shorter compared to guts from wild-type flies 48 hours postinfection (Figure 6c). Altogether, we conclude that the EGFR pathway regulates cell morphogenesis during enterocyte maturation and that this process is critical during normal development of the adult gut, compensatory renewal following infection and aging.

### The EGFR pathway is required to coordinate delamination and anoikis of enterocytes during infection

Our results support a role of the EGFR pathway in both ISC proliferation and the shaping of enterocytes. Surprisingly, the guts of flies with reduced levels of EGFR or Ras activity in enterocytes did not decrease in length and increase in width to the same extent as the guts of wild-type flies (Figure 6c), suggesting a role for the EGFR pathway in cell sloughing.

**Figure 7:**
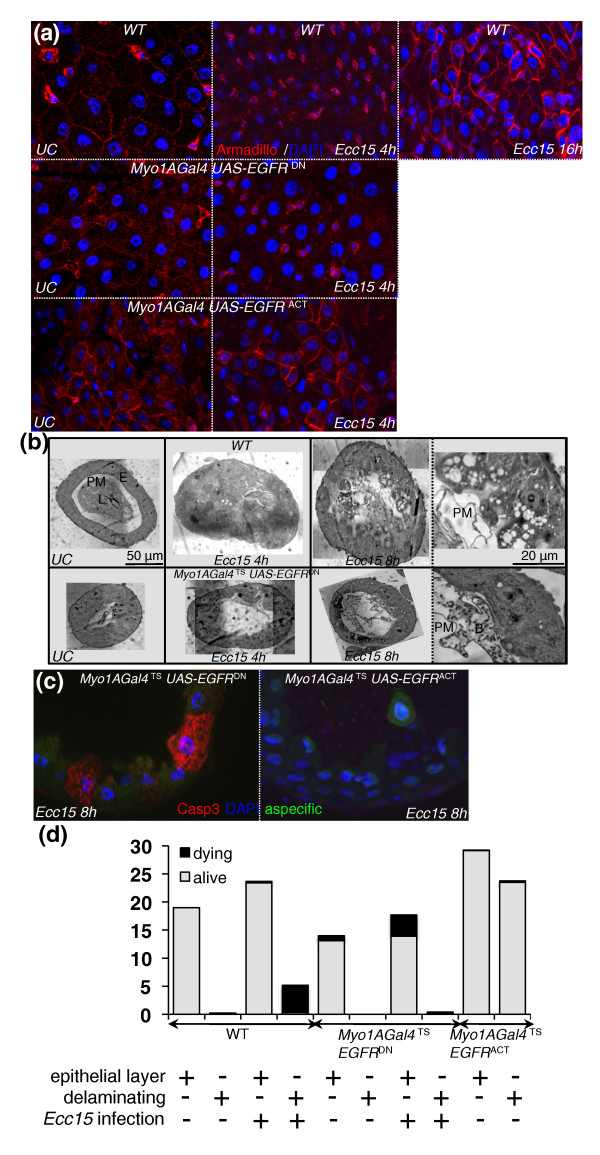
**The EGFR pathway is required for enterocyte delamination upon infection**. **(a) **Immunostaining of gut epithelium with Armadillo (red) and DAPI (blue). In WT flies, the Armadillo signal was strong in progenitors and lower in enterocytes. Upon infection, Armadillo staining disappeared from enterocyte membranes, but remained intense in progenitor cells. At later stages, both progenitors and newly synthesized enterocytes displayed a strong Armadillo signal. Guts with enterocytes depleted of EGFR activity (*UAS-EGFR^DN^*) displayed low Armadillo staining in enterocytes that did not change upon infection. In the absence of infection, guts with activated EGFR (*UAS-EGFR^ACT^*) in enterocytes exhibited a pattern of Armadillo staining similar to *Ecc15*-infected WT guts. **(b) **Histological sections of guts from flies with WT (top) and EGFR-depleted enterocytes (bottom) with and without infection. A strong multilayering of epithelial cells (*t *= 4 hours), followed by their blebbing, and delamination (*t *= 8 hours) are observed in WT guts following infection. Delaminating cells contain multiple large vacuoles. In contrast, the multilayering, blebbing and delamination of enterocytes was not observed in guts from flies depleted of EGFR in enterocytes. PM, peritrophic matrix; L, lumen; E, enterocyte; B, bacteria. **(c) **In contrast to WT (Figure 2e), apoptotic enterocytes (anticleaved caspase 3-positive) were detected within the epithelial layer in flies with enterocytes depleted of EGFR activity (*UAS-EGFR^DN^*). Neither delaminated cells nor enterocytes within the epithelium were apoptotic in guts with activated EGFR (*UAS-EGFR^ACT^*) in enterocytes. **(d) **Quantification of delaminating and apoptotic cells in the guts of flies with WT enterocytes, enterocytes depleted of EGFR activity (*UAS-EGFR^DN^*) or enterocytes expressing an activated form of EGFR (*UAS-EGFR^ACT^*). Cells within the epithelium layer and delaminating cells were counted from three histological sections (*N *= 16 guts) for each genotype in UC and *Ecc15*-infected flies. The proportions of living and dead cells per section were determined with anticleaved caspase 3 and DAPI staining.

We first investigated the role of the EGFR pathway in epithelial delamination by analyzing the dynamics of adherens junction in the gut during infection. E-cadherin is a component of adherens junctions and serves as the key mediator of epithelial cell-to-cell adhesion. Immunostaining against Armadillo (β-catenin), an intracellular partner of E-cadherin, revealed that Armadillo was strongly expressed around progenitor cells and present at the cell membrane of enterocytes (Figure 7a) [4,20]. However, at 4 hours postinfection, Armadillo staining was less homogeneous and frequently appeared punctate and localized to the cytoplasm (Figure 7a). The loss of membrane staining occurred mainly in mature enterocytes, defined as cells with large, highly polyploidized nuclei, which were the most apically positioned within the epithelial layer (Additional file 13A). Accordingly, E-cadherin was also internalized in some enterocytes of infected flies (Additional file 13). The destabilization and relocalization of Armadillo and E-cadherin are indicative of a disruption of adherens junctions in mature enterocytes and likely reflect a weak attachment between cells, such as would occur during the detachment and sloughing of cells from the epithelium. Between 4 and 16 hours postinfection, strong Armadillo staining was observed around ISCs and newly synthesized enterocytes (Figure 7a). Interestingly, the pattern of Armadillo staining did not change during infection in flies depleted of EGFR in enterocytes, but instead a weak and constant Armadillo staining was observed in enterocytes. Nevertheless, wild-type Armadillo staining was observed around progenitor cells of flies with reduced EGFR in enterocytes (Figure 7a). In contrast, Armadillo staining in the guts of unchallenged flies expressing an activated form of EGFR in enterocytes was similar to the nonhomogeneous pattern that we observed in wild-type infected flies, displaying both young enterocytes with strong Armadillo staining and mature enterocytes with no Armadillo staining (Figure 7a). Finally, clones of enterocytes expressing an active form of Ras lose Armadillo staining at their membrane (Additional file 13D). Collectively, this suggests that the EGFR pathway modulates adherens junction dynamics in enterocytes during epithelial renewal.

To investigate further the role of EGFR in the delamination process, we examined histological sections of guts upon infection. In contrast to wild-type flies, we did not observe cell blebbing, the multilayering of enterocytes or loss of cells into the lumens of flies depleted for EGFR in enterocytes (Figure 7b and Additional files 3and 4). The lack of cell sloughing was further confirmed by a strong reduction of aspecific yellow fluorescence in the guts of these flies (Additional file 4C). We previously reported that in wild-type flies enterocytes undergo apoptosis only in the lumen following detachment from the epithelium layer (Figure 2e). In sharp contrast, apoptotic enterocytes with fragmented nuclei were observed within the epithelium of flies depleted of EGFR in enterocytes, as revealed by caspase 3 activity and 4',6-diamidino-2-phenylindole (DAPI) staining (Figure 7c and quantification in Figure 7d). Conversely, the overexpression of the EGFR pathway in enterocytes induced high levels of cell delamination in the absence of infection (Figure 7d and Additional files 3 and 4A). Accordingly, clones of enterocytes expressing activated forms of EGFR or Ras (*UAS-EGFR^ACT ^*and *UAS-Ras^v12^*, respectively) rapidly disappeared from the epithelium compared to wild-type clones, suggesting an increased rate of delamination (Additional file 4). Similarly, overexpression of the EGFR ligand Keren resulted in a thick, multilayering of the gut due to the accumulation of cells in the lumen (Additional file 3). The detached cells in these guts differed from those in wild-type flies in that they did not undergo cell death, had normal nuclei and did not contain enlarged vacuoles (Figure 7c and quantification in Figure 7d).

These results indicate multiple roles of the EGFR pathway in enterocytes in response to infection, where it is required for early cell blebbing, modulation of cell junction dynamics and the normal process of enterocyte death. We conclude that a wild-type level of EGFR pathway activity is required in enterocytes for the proper coordination of their delamination and anoikis upon infection.

## Discussion

To date, studies on epithelium renewal have mainly focused on stem cell activity and its role in basal epithelium maintenance [17-19]. Recently, several studies have shown that ISC activity is strongly stimulated in response to damage-inducing agents and infectious bacteria [6,8,9,11,21]. Importantly, these stimuli induce ISC activity to an extent that facilitates the identification of underlying regulatory networks. Using this approach, we have performed quantitative analysis of gut remodeling at different time points following infection with *Ecc15*. We demonstrate that restoration of normal gut morphology involves more than the activation of ISCs, but rather requires the coordination of ISC proliferation and differentiation, the incorporation and morphogenesis of new cells and the expulsion of damaged enterocytes. We further demonstrate that the EGFR pathway is central to these three steps following bacterial infection.

### Epithelium renewal: A more complex picture

Our quantitative analysis of distinct cell populations in the gut identified two phases of compensatory repair that occur following bacterial infection. Initially, the pool of preexisting precursor cells (enteroblasts) rapidly differentiate to buffer the loss of cells. This first phase does not require ISC proliferation and likely serves the same function as the process of epithelium restitution described in mammals, where neighboring cells migrate into damaged regions to maintain gut integrity [22]. This early step is followed by a longer, regenerative phase involving ISC proliferation and differentiation, which is capable of repairing the entire gut in approximately 2 days. The precision with which the gut is regenerated is striking, returning to its original dimensions within a small percentage of variation.

Our quantitative analysis also revealed the striking regenerative capacity of the gut, which maintains its integrity despite the loss of nearly half of the enterocytes and a 40% reduction of its size. This massive cell loss is first observed as a multilayering of enterocytes that contributes to the characteristic increase in gut width observed upon infection. Subsequently, cells are expelled into the lumen. These detached enterocytes appear highly vacuolized and undergo cell death only when they have left the epithelium. This process is similar to anoikis, a mechanism of cell death induced by the loss of cell adhesion that occurs in the guts of mammals [23]. While the delamination of cells correlates temporally with the drastic shortening of the gut, we cannot exclude the involvement of additional processes. One hallmark of the response of the mammalian gut to infection is the contraction of visceral muscles [24]. The contribution of visceral muscles to gut shrinking in *Drosophila *remains to be determined. Globally, the combination of the gut shortening with cells dying after leaving the epithelium through anoikis provides an efficient mechanism to maintain gut integrity and may explain its ability to resist infection and the action of damaging agents.

### The EGFR and JAK/STAT pathways are both required for ISC proliferation

**Figure 8:**
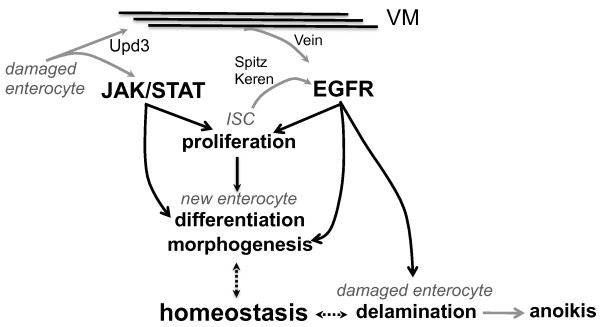
**Regulation of epithelium renewal by the JAK/STAT and EGFR pathways**. Upon infection, damaged enterocytes release Upd3. This cytokine activates the JAK/STAT pathway in both progenitor cells (ISCs) and in the surrounding visceral muscles (VMs). JAK/STAT activation in VMs participates in the induction of the EGF *vein*. In addition, two EGFs, Keren and Spitz, are also secreted by the epithelium. The activation of both the JAK/STAT and EGFR pathways in progenitor cells stimulates their proliferation. The JAK/STAT pathway has a unique role in enteroblast differentiation, while the EGFR pathway is required in enterocytes for proper morphogenesis and delamination of damaged enterocytes following infection with *Ecc15*. Other ligands of the JAK/STAT pathway (Upd1 and Upd2) may also participate in bacteria-induced ISC proliferation.

In this study, we show that a canonical EGFR pathway is required in ISCs to promote their proliferation. Reduction of the EGFR pathway in ISCs does not affect the maintenance of ISCs in short term, but prevents their activation in response to damage caused by infection. As a consequence, flies with reduced EGFR activity in ISCs failed to repair their guts following infection, and they died within 10-14 days. We further show that EGFR is activated in ISCs by three ligands, Vein, Keren and Spitz. Vein is produced by the visceral muscles in response to infection, whereas Keren and Spitz are produced within the epithelium. This is similar to the scenario in larvae, where Vein is produced by visceral muscles and is required for adult midgut progenitor proliferation, while at later time points the progenitors themselves express Spitz and Keren [16]. In the adult gut, Vein is secreted by the inner layer of circular visceral muscles, but not by the outer layer of longitudinal visceral muscles. Intriguingly, ISCs are in very close contact with these inner visceral muscles (Additional file 14). Moreover, *vein *is partially regulated by the JAK/STAT pathway in visceral muscles in response to the production of the *Upd3 *cytokine by damaged enterocytes. We hypothesize that, because of this close association, visceral muscles may act as a connective tissue to relay signals between damaged enterocytes and distant ISCs. Such a system would act to spatially and temporally restrict EGFR activation to the segment around the damaged area in which proliferation, morphogenesis and delamination would then be synchronized (see model in Figure 8).

Our epistatic analysis indicates that both the EGFR and JAK/STAT pathways are required to promote ISC proliferation. However, inhibition of the EGFR pathway completely blocks proliferation induced by overexpression of either Upd3 or Domeless in ISCs, whereas JAK/STAT depletion in ISCs only partially reduces the proliferation induced by activation of the EGFR pathway. This suggests a more central role for the EGFR pathway in ISC proliferation. Since our epistatic analysis involved partial loss of function of genes as a result of the use of RNAi and dominant negative proteins, it is difficult to establish a hierarchy between these two pathways. Future studies should address the respective roles of these pathways in ISCs, as well as the mechanisms by which they induce ISC proliferation. Of note, synergy between these two pathways has already been described in *Drosophila*, where both STAT and Ras are required in germ cells and tumor cells to promote cell proliferation and migration [25,26]. Moreover, mild expression of the proto-oncogene *Ras^V12 ^*in the gut promotes a preoncogenic state that, in combination with infection, induces dysplasia [21]. An implication of the EGFR pathway in ISC proliferation is supported by a very recent study [27] that shows that the EGFR and JAK/STAT pathways are required in ISCs for the proliferation induced by the lack of Hippo signaling in enterocytes. Although both are required for ISC proliferation, the JAK/STAT and EGFR pathways also have distinct functions: the JAK/STAT pathway is required for enteroblast differentiation, while the EGFR pathway is required for proper gut morphogenesis and cell sloughing.

### A dual role of EGFR in enterocytes

Our study points to a dual role of the EGFR pathway in the morphogenesis and sloughing of enterocytes. Flies with reduced EGFR activity in enterocytes have a characteristic long and thin gut that results from the flattening of enterocytes (scheme Additional file 11D), indicating that aberrant cellular morphogenesis has repercussions on the morphology of the tissue as a whole. The requirement of the EGFR pathway for enterocyte shape appears to occur during the maturation of newly synthesized enterocytes and affects at least three different morphogenetic events in the gut: the initial development of the adult gut, the basal maintenance upon aging and the accelerated renewal that occurs in response to damage induced by infection. In agreement, the guts of flies with reduced EGFR signaling in progenitors (*esgGAL4^TS^*; *UAS-EGFR^DN ^or Su(H)GBEGal4*; *UAS-EGFR^DN^*) are longer, indicating that EGFR modulates enterocyte shape at the late progenitor to young enterocyte transition (Additional file 15). The effect of the EGFR pathway on cell morphogenesis is supported by previous work reporting that EGFR affects tracheae and wing vein remodeling through its impact on E-cadherin-based cell adhesion [28-30]. A role of EGFR in enterocyte-to-enterocyte adhesion is also suggested by our observation that E-cadherin and Armadillo change their subcellular localization during epithelium renewal. Along with these studies, our results point to a general role of EGFR in epithelium morphogenesis. It would be interesting to investigate whether differences in the level of EGFR signaling determine the type of epithelia, squamous or columnar, encountered along the gut. In this sense, we observed that the largest increase in the gut length of flies defective in EGFR is mostly due to the intense flattening of cells in a region of the gut that is normally highly folded and composed of columnar enterocytes. Interestingly, our survival analysis points to a key role of EGFR-mediated enterocyte morphogenesis in the maintenance of the integrity of the gut, as shown by the increased susceptibility of EGFR-deficient flies to infection.

Surprisingly, we observed that altering the EGFR pathway activity in enterocytes strongly affected the delamination process. We observed that, upon infection, reduction of EGFR pathway activity in enterocytes decreased the sloughing of cells from the epithelium and led to apoptosis of enterocytes still within the layer. Conversely, expression of an activated form of this receptor, stimulated cell sloughing, but subsequent enterocyte death was not observed. We conclude that the EGFR pathway is essential for the proper execution of anoikis in response to infection. The presence of dead cells within the epithelium and reduced delamination could explain the disruption of gut barrier integrity observed in flies with reduced EGFR activity in enterocytes. Our data are compatible with the idea that EGFR pathway provides a transient survival signal to damaged cells, enabling them to delaminate and leave the epithelium before dying. This prosurvival effect of EGFR would explain why the overexpression of the EGF ligand Keren is sufficient to induce cell delamination, but there is no consecutive apoptosis. It is tempting to speculate that the loss of accessible growth factors such as EGFs by delaminated cells ends this transient survival period and switches the cell to enter anoikis. However, it remains unclear whether the function of EGFR in enterocyte delamination is dependent or not of EGFR ligands. We also observed a clear role of EGFR in the remodeling of the adherens junction during epithelium renewal. These findings are consistent with a number of studies highlighting the role of this pathway in anoikis in mammals. Early loss of E-cadherin from cell-to-cell junctions is involved in the onset of anoikis in human enterocytes [31]. Moreover, using an *in vitro *model of culture of villus epithelium, Lugo-Martínez *et al. *[32] observed that inhibition of EGFR maintains E-cadherin at the membrane and decreases anoikis in mammals. We similarly observed a modulation of E-cadherin and Armadillo localization in the *Drosophila *gut during epithelium renewal, suggesting that a disassembly of E-cadherin-mediated junctions occurs during cell detachment. In agreement with this observation, overexpression of cadherin or dlg in enterocytes reduces the gut shortening normally observed with infection (Additional file 13E). Future studies should investigate whether EGFR mediates its effect through the disassembly of E-cadherin adherens junctions and what downstream components of the pathway are involved. In addition, it is not yet clear whether loss of E-cadherin is a cause or a consequence of cell detachment. In our study, we cannot exclude the possibility that the effects of EGFR on cell sloughing could also be a consequence of the abnormal morphogenesis of newly synthesized enterocytes, whose growth may aid in physically pushing damaged enterocytes out of the layer. Alternatively, new enterocytes may require the space created by delaminating enterocytes to shape properly. These scenarios are not mutually exclusive, and it is possible that the EGFR pathway separately modulates the integration of new enterocytes into the epithelia and their elimination by sloughing, thereby controlling the flux of intestinal cells.

Importantly, the implication of EGFR in three crucial stages of epithelium renewal, ISC proliferation, the synthesis and morphogenesis of new enterocytes and the elimination of damaged cells, could explain the synchronization of the complex cellular events that maintain gut homeostasis. The release of signals from damaged enterocytes that promote ISC proliferation and differentiation, in addition to enterocyte morphogenesis and elimination, provides a homeostatic loop coordinating gut repair. In this line, the cytokine Upd3 is a good candidate because it is produced by damaged enterocytes and capable of activating both the JAK/STAT and EGFR pathways, directly or through the production of Vein in muscles (Figure 8).

## Conclusions

Our data provide new insights into the complex events regulating gut remodeling upon infection. Our cellular analysis identified striking similarities between the *Drosophila *and mammalian gut epithelium response to damage and suggests the conservation of some regulatory networks. Interestingly, stimulation of stem cell activity by invasive, bacteria-like *Salmonella *has been demonstrated to induce proliferation in mammalian guts [33], and gut infection is proposed to favor the development of cancer [34,35]. Our study provides potential mechanisms to explain these links. Moreover, the EGFR pathway is involved in the maintenance of gut barrier integrity in mammals and is important in preventing the development of colitis [36]. Thus, the use of infection to study *Drosophila *ISCs provides not only a powerful model to dissect stem cell regulation but also to elucidate the complex mechanisms that maintain tissue homeostasis and gut morphogenesis.

## Methods

### Fly stocks

Oregon^R^, Canton^S^, flies or flies carrying one copy of the *Myo1A-Gal4*, *how-Gal4 *or *esg-Gal4 *transgene were used as wild-type controls. A complete list of stocks is provided in supplementary materials (Additional file 16 and associated references [17,20,37-47]). For experiments, we used adult flies carrying one copy of the UAS construct (RNAi or dominant negative (DN)) combined with one copy of the Gal4 driver. The F1 progeny carrying both the UAS construct and the Gal4 driver were raised at 18°C until 3 days of adult development and then transferred to 29°C for optimal efficiency of the UAS/GAL4 system. *Drosophila *stocks were maintained at 23°C using standard fly medium (maize flour, dead yeast, agar and fruit juice) devoid of living yeast. Conditional *esgGal4^TS^*, *howgal4^TS ^*or *Myo1AGal4^TS ^*animals were obtained by crossing virgin females (*esg/how/Myo1A-Gal4*, *UAS-GFP*; *tub-Gal80^TS^*) with males expressing a UAS construct. F1 progeny were raised at 18°C, and the activity of the Gal4 system was controlled by placing 3-day-old F1 adults at either restrictive (29°C, Gal80^TS ^off, Gal4 system on) or permissive (18°C, Gal80^TS ^on, Gal4 system off) temperatures. The clonal labeling of mature enterocytes was performed by shifting flies of the genotype *yw,hsFLP;act_FRT_yellow_FRT_Gal4*, *UAS-GFP/CyO *to 29°C for 1 day. At this temperature, the basal FLP expression is enough to enable flipout events in enterocytes (during their polyploidization) and GFP induction. To induce somatic recombination in flies expressing the mosaic analysis with a repressible cell marker system [48] or *yw,hsFLP;act_FRT_yellow_FRT_Gal4, UAS-GFP/CyO*, 3-day-old adult flies were heat-shocked for 60 minutes at 37°C for 3 consecutive days. Three days after or 1 week after, guts were dissected for immunostaining. Adherens junctions were marked by a *DE-cadherin-GFP *fusion protein expressed ubiquitously under the control of the *ubi *promoter [20]. Septate junctions were marked by a *Dlg-GFP *construct expressed under the control of its own promoter [49]. Autophagy markers were expressed under the control of the *Myo1A-Gal4 *driver and are described elsewhere [46].

### Bacterial strains and infection experiments

*E. carotovora carotovora 15 *is a Gram-negative bacterium that induces a strong local immune response [8] and is described elsewhere [50]. For oral infection, 3-day-old to 5-day-old flies were incubated for 2 hours at 29°C in an empty vial before being transferred to a fly vial with infection solution and maintained at 29°C. The infection solution was obtained by mixing an equal volume of 100× concentrated pellet from an overnight culture of *Ecc15 *(optical density OD_600 _= 200) with a solution of 5% sucrose (1:1) and deposited on a filter disk that completely covered the surface of standard fly medium. Flies were incubated for 1 day at 29°C on the contaminated filter, after which they were transferred to fresh vials. In the case of survival analysis, flies were continuously exposed to filters contaminated with *Ecc15 *and flipped every 2 days into new vials containing a filter contaminated with a fresh pellet of *Ecc15*. Survival was monitored every day.

Quantification of *Ecc15 *was determined from three individual replicates of five flies at 0.5, 1, 2, 4, 8, 12, 24 and 48 hours following infection with *Ecc15*. Dissected midguts were placed in 1 mL of phosphate-buffered saline (PBS) in a 1.5-mL screw top microcentrifuge tube containing glass beads. The samples were homogenized using a Precellys 24 (Bertin Technologies, France), and then dilutions were plated on Luria Broth Agar (MP biomedicals, Illkirch, France) and incubated at 29°C. Colonies were counted after 24 hours. For hemolymph colony-forming unit counts, flies were surface sterilized in 70% ethanol and pricked in the thorax (once per side) with a thin sterile needle. The exuding drops of hemolymph were collected with a pipette and transferred to a 0.5-mL microcentrifuge tube for dilution plating as described above.

### Immunostaining

For live imaging, guts were dissected in PBS and immediately mounted in the antifading agent Citifluor AF1 (Citifluor Ltd, London, UK). For immunofluorescence, guts were dissected in PBS, fixed for 20 minutes in 0.1% Tween 20-PBS (PBT) with 4% paraformaldehyde, rinsed in PBT and then incubated with primary antibodies (dilution 1:50 anti-Armadillo (Developmental Studies Hybridoma Bank, Iowa city, Iowa, USA), 1:500 anti-PH3 (Millipore, Billerica, Massachusetts, USA), 1:500 anti-β-galactosidase (Promega, Madison Wisconsin, USA), 1:300 anti-phospho-ERK (in tributyltin; Cell Signaling, Boston, Massachusetts, USA), 1:500 anti-phospho-JNK (Cell Signaling), 1:500 anticleaved caspase3 (Cell signaling) and 1:1,000 anti-GFP (Roche, Rotkreuz, Switzerland)) or Rhodamine-Phalloidin (dilution 1:50 (Invitrogen, Basel, Switzerland)) in PBT + 1% bovine serum albumin. Primary antibodies were revealed with Alexa488- or Alexa594-coupled antimouse antibodies (Invitrogen), and nuclei were stained with DAPI (Sigma, Saint Louis, Missouri, USA). Guts were then scanned with an Axioplot imager (Zeiss, Feldbach, Switzerland) and recomposed using the program MosaiX (Zeiss).

### Morphological analysis

Measurements in Figure 1a and Additional files 1 and 2 were determined from dissected guts fixed as described above and stained with DAPI. Individual guts (*N *= 20 to 40) were visualized and captured using an Axioplot imager (Zeiss). Full guts were scanned at ×10 magnification and then recomposed with MosaiX (Zeiss). Images from representative fields of the same guts were captured at ×20 magnification in Z-stacks, and full projections were counted. Measurements to the nearest micron were obtained using the measure functions within AxioVision software (Zeiss, Feldbach, Switzerland). Length was measured by tracing from the middle of the proventriculus along the midgut to the midgut-hindgut junction (indicated by the branching of the Malphigian tubules). Gut width measurements are based on the average of five measurements taken along the gut. Counts of midgut cells were estimated on the basis of values obtained from counting the different cell population in the projected Z-stack images and then multiplying the sum to the area of the whole gut. The distinction between cell types was based on GFP staining (progenitors expressing GFP under the control of the *esgGal4 *driver) and the level of polyploidy (nuclear size). New enterocytes were defined as having low polyploidy (intermediate-sized nuclei) and/or persisting GFP signal (due to residual *escargot-GFP *signal), while old enterocytes were defined as having high polyploidy (large-sized nuclei). To verify the estimated cell counts, full counts were conducted of each cell type from five wild-type guts. Cell density was determined by measuring the distance from the nucleus of a given cell to the nucleus of its nearest neighboring cell, or distance internuclei (DIN). The values for 20 cells in a single field per gut of *N *= 20 guts were measured. The same position in the gut was recorded each time, and the average distance for each genotype was plotted. Delamination was quantified by counting the number of attached cells (within the epithelium layer) or unattached cells (outside the epithelium layer) in histological sections (see below). The values of three sections of *N *= 16 guts were measured for each genotype with and without infection. For all measurements, similar regions were recorded and all samples were exposed to identical conditions.

### Histological sections

*Drosophila *adults were dissected into PBS, and the guts were immediately fixed with 2.5% glutaraldehyde and 2% paraformaldehyde in PBS for 4 hours at 4°C. The samples were rinsed three times in 0.1 M cacodylate buffer, then postfixed with 1% osmium tetroxide and 1.5% potassium ferrocyanide solution in 0.1 M cacodylate buffer for 40 minutes at room temperature, followed by 1% osmium tetroxide solution in 0.1 M cacodylate buffer for 40 minutes at room temperature. The samples were then treated with 1% uranyl acetate in water for 40 minutes at room temperature. Dehydration of the guts was performed in an ascending series of ethanol concentrations, and then the samples were embedded in Durcupan (Sigma). The guts were cut at 0.2 mm (semithin sections) for light microscopy with a Leica ultramicrotome (Leica, Wetzlar, Germany). Semithin sections were stained with 2% toluidine blue and observed under a Zeiss microscope. For ultrathin sections, guts were cut at 50 nm for transmission electron microscopy with a Leica ultramicrotome. Ultrathin sections were contrasted with lead citrate and observed with an electron microscope. Immunostaining on cryostat sections was performed using the protocol described by Baumann [51].

### RT-qPCR

Total gut RNA was extracted from 30 dissected midguts with TRIzol reagent (Invitrogen). Template RNA (1 μg) was used to generate cDNA by reverse transcription and then analyzed by quantitative polymerase chain reaction (qPCR) with a LightCycler 2.0 and the SYBR Green I kit (Roche). Expression values were normalized to *RpL32*. Primers used to monitor mRNA quantification can be obtained upon request.

### Statistical analysis

Mean fly mortality, mean mitosis per gut (PH3 counts) and mean relative gene expression and their corresponding standard errors were determined using PROC MEANS (SAS Institute, Cary, NC, USA). Means were separated for significance using Fisher's protected least significant difference test at *P *= 0.05.

## Abbreviations

DN: dominant negative; *Ecc15*: *Erwinia carotovora carotovora 15*; EGF: epidermal growth factor; EGFR: EGF receptor; ERK: extracellular signal-regulated kinase; ISC: intestinal stem cell; JAK/STAT: Janus kinase-signal transducers and activators of transcription; MAPK: mitogen-activated protein kinase; PH3: phosphohistone H3.

## Authors' contributions

NB, NAB and BL conceived and designed the experiments. NB, NAB and TK performed the experiments. NB, NAB and BL analyzed the data. NB, NAB and BL wrote the paper. All authors read and approved the final manuscript.

## Supplementary Material

Additional file 1 **Measurements of several parameters in the *Drosophila *gut following infection with *Ecc15***. Click here for file

Additional file 2 **Dynamics of the different cell populations in the midgut following infection with *Ecc15***. Click here for file

Additional file 3 **Transversal sections of guts from wild-type flies and flies with an altered level of EGFR pathway activity in enterocytes**. Click here for file

Additional file 4 **The EGFR pathway is required for enterocyte delamination upon infection**. Click here for file

Additional file 5 **Enterocytes undergoing delamination display marks of increased autophagy**. Click here for file

Additional file 6 **The EGFR pathway is required in ISCs for the ISC proliferation induced by infection**. Click here for file

Additional file 7 **Flies with a reduced level of EGFR pathway activity in ISCs display an increased susceptibility to *Ecc15 *infection**. Click here for file

Additional file 8 **The EGFR ligands Spitz and Keren are expressed in the progenitor cells**. Click here for file

Additional file 9 **The JAK/STAT pathway is activated in the visceral muscles**. Click here for file

Additional file 10 **EGFR and JAK/STAT pathways synergize in ISCs to promote proliferation**. Click here for file

Additional file 11 **Alteration of the EGFR pathway in enterocytes affects gut morphology**. Click here for file

Additional file 12 **Flies with reduced EGFR activity in enterocytes are highly susceptible to oral infection with *Ecc15***. Click here for file

Additional file 13 ***Ecc15 *infection alters adherens junction dynamics in the gut**. Click here for file

Additional file 14 **ISCs are in close proximity to visceral muscles**. Click here for file

Additional file 15 **The EGFR pathway is required in both enteroblasts and enterocytes for proper morphogenesis**. Click here for file

Additional file 16 **List of fly stocks used in this study**. Click here for file
