# Supplementary material for: Drosophila EGFR pathway coordinates stem cell proliferation and gut remodeling following infection
Source: BMC Biol. 2010 Dec 22;8:152. doi: 10.1186/1741-7007-8-152 (PMC3022776; doi:10.1186/1741-7007-8-152)
Supplement: Additional file 1 — Measurements of several parameters in the Drosophila gut following infection with Ecc15. [file 1741-7007-8-152-S1.PDF]

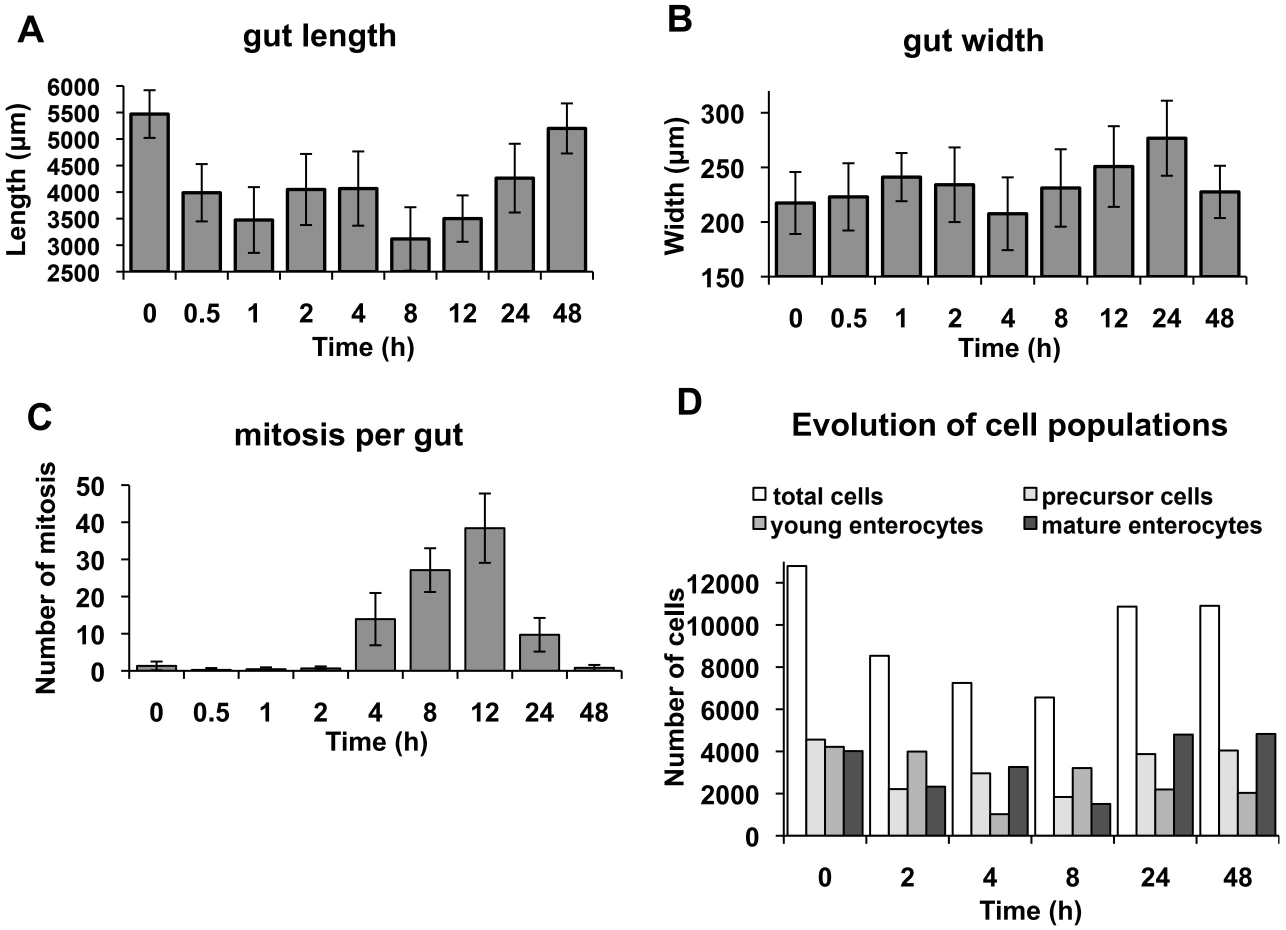

Additional File 1. Measurements of several parameters in the *Drosophila* gut following infection with *Ecc15*. **(A-B)** Variations in gut length and width (in μm) of wild-type flies following infection with *Ecc15.* Dissected guts were stained with DAPI and single images were compiled from 10X scans of individual guts using the MosaiX feature of AxioVision (Zeiss). Midgut measurements were made using the AxioVision image analysis software (Zeiss). Cell counts per gut were estimated from three representative fields (20X) performed in the anterior, middle and posterior midgut of five guts for each time point and condition. Full counts of five individual midguts were performed to verify the estimates of the number of cells. **(C)** The number of mitotic stem cells was determined by counting PH3-positive cells along the midgut. Measurements were based on five replicates (N=10 guts) for each time point. **(D)** Variation in cell populations in the gut of wild-type flies upon *Ecc15* infection. The proportion of mature enterocytes (cells with large nuclei due to high degree of polyploidy), young enterocytes (cells with medium-sized nuclei due to low degree of polyploidy and GFP-positive due to the persistence of the *escargot-GFP* marker), and progenitors (cells with small nuclei and strongly labeled with *escargot-GFP* marker) were counted from dissected guts stained with DAPI.
