# Supplementary material for: Drosophila EGFR pathway coordinates stem cell proliferation and gut remodeling following infection
Source: BMC Biol. 2010 Dec 22;8:152. doi: 10.1186/1741-7007-8-152 (PMC3022776; doi:10.1186/1741-7007-8-152)
Supplement: Additional file 2 — Dynamics of the different cell populations in the midgut following infection with Ecc15. [file 1741-7007-8-152-S2.PDF]

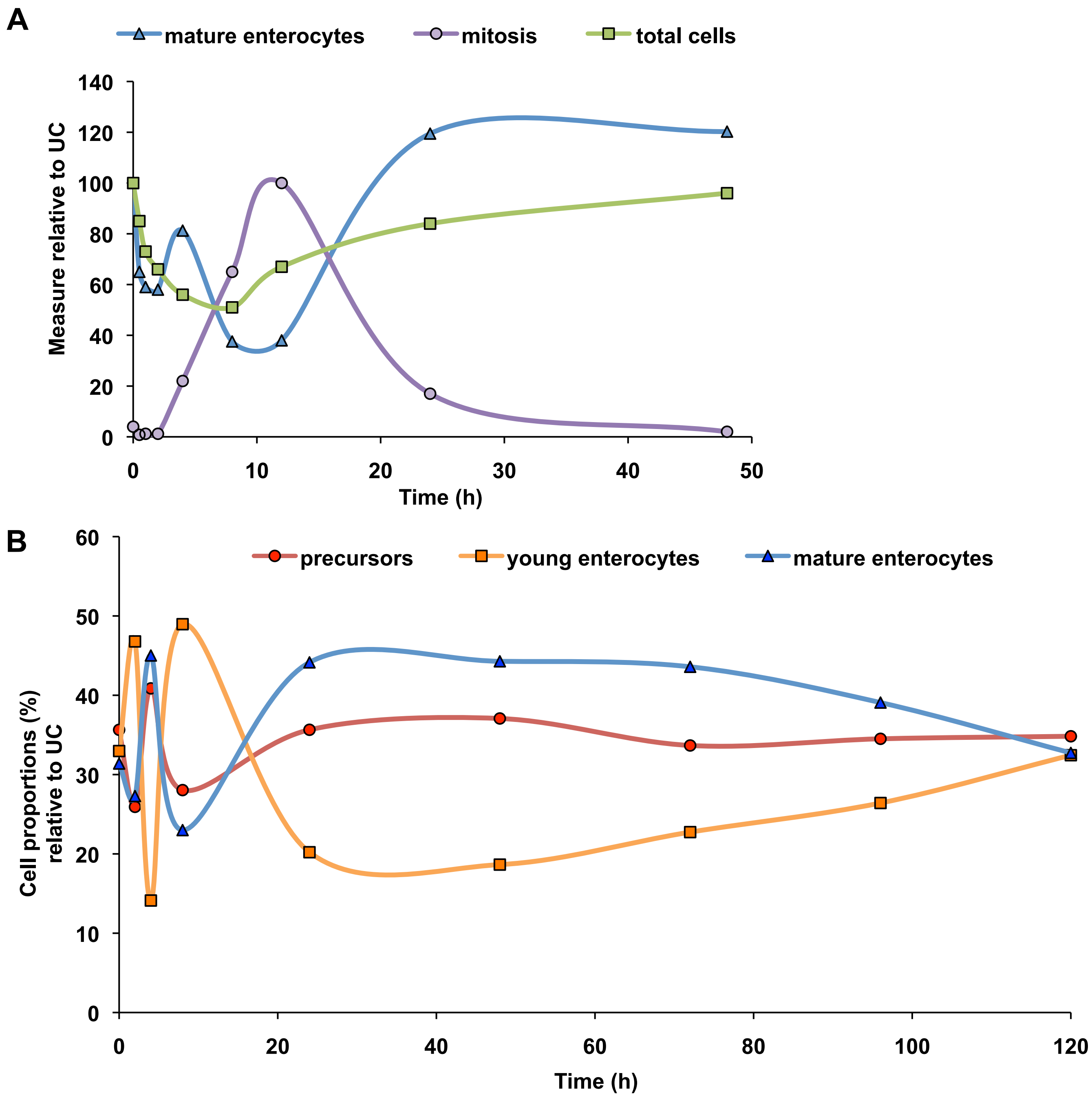

Additional file 2. Dynamics of the different cell populations in the midgut following infection with *Ecc15*.
(A) Quantitative measurements of the number of total cells and mature enterocytes at different time points following ingestion of *Ecc15*, shown relative to counts in unchallenged (UC) midguts. The number of mitotic events (measured as PH3-positive cells) at each time point is also shown. **(B)** Proportion of each cell type at different time points following infection. The different cell types were counted as described in methods and Additional file 1D.
