# Supplementary material for: Drosophila EGFR pathway coordinates stem cell proliferation and gut remodeling following infection
Source: BMC Biol. 2010 Dec 22;8:152. doi: 10.1186/1741-7007-8-152 (PMC3022776; doi:10.1186/1741-7007-8-152)
Supplement: Additional file 3 — Transversal sections of guts from wild-type flies and flies with an altered level of EGFR pathway activity in enterocytes. [file 1741-7007-8-152-S3.PDF]

A

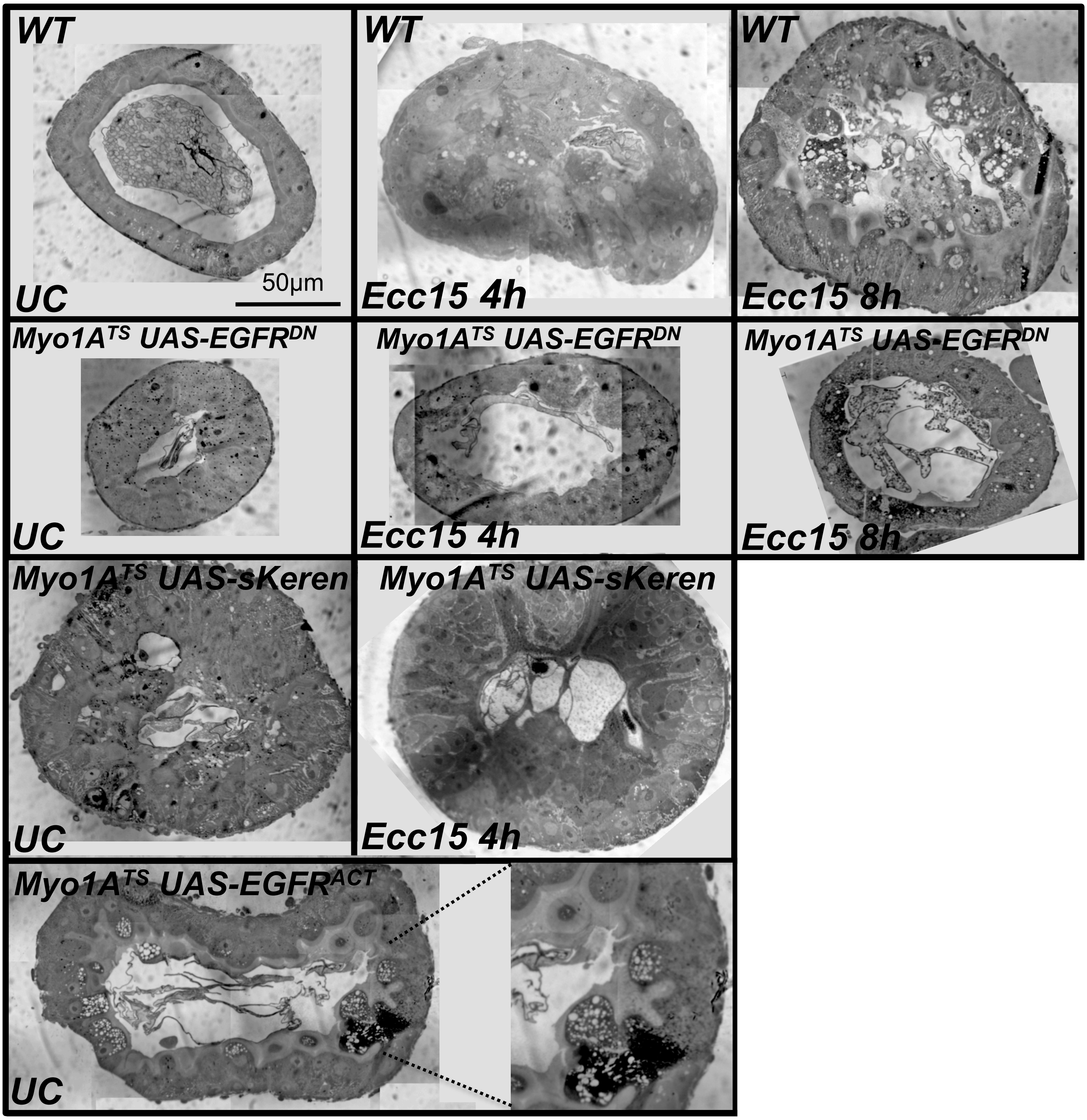
WT
UC
50μm
WT
Ecc15 4h
WT
Ecc15 8h
Myo1A^TS UAS-EGFR^DN
UC
Myo1A^TS UAS-EGFR^DN
Ecc15 4h
Myo1A^TS UAS-EGFR^DN
Ecc15 8h
Myo1A^TS UAS-sKeren
UC
Myo1A^TS UAS-sKeren
Ecc15 4h
Myo1A^TS UAS-EGFR^ACT
UC

B

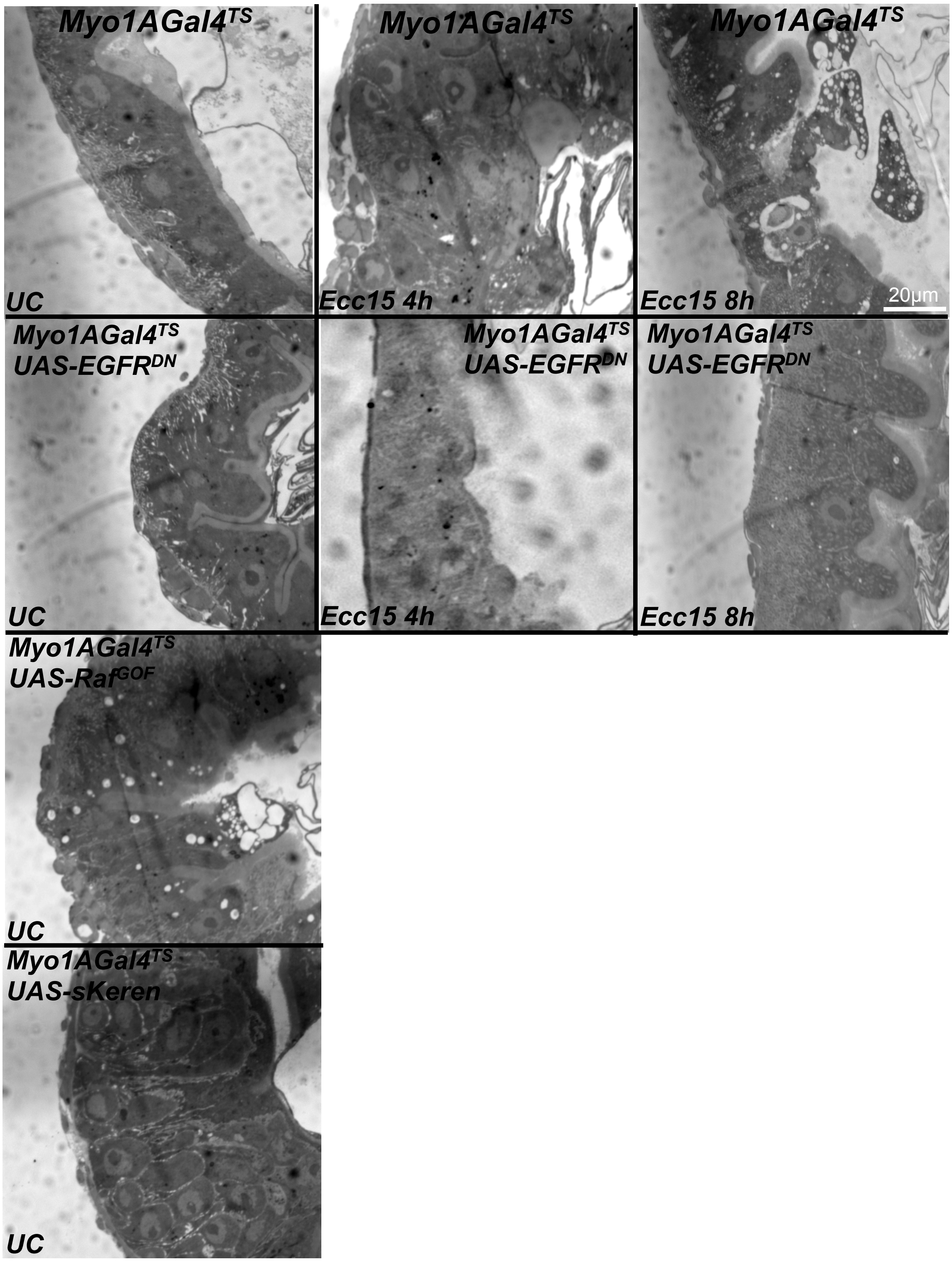

Additional file 3. Transversal sections of guts from wild-type flies and flies with an altered level of EGFR pathway activity in enterocytes.

(A) Representative images of histological sections of guts from flies with wild-type enterocytes (WT), EGFR-depleted enterocytes (*Myo1AGal4^{TS} UAS-EGFR^{DN}*), or EGFR-activated enterocytes (*Myo1AGal4^{TS} UAS-EGFR^{ACT} or UAS-sKeren),* with and without infection are shown. Multilayering (t=4 h) and delamination (t=8 h) of epithelial cells is observed in wild-type guts following infection. Delaminating cells contain large vacuoles indicative of lysis. In contrast, multilayering and cell delamination were not observed in guts from flies depleted for EGFR in enterocytes. Flies with enterocytes expressing an activated form of EGFR or a secreted form of Keren display multilayered gut epithelia in absence of infection. In these flies, only a few (EGFR activated, inset) to none (sKeren) of the delaminating cells undergo apoptosis. The multilayering of cells was more marked with sKeren probably due to the fact that this diffusible EGF ligand can also promote ISC proliferation when it is expressed in enterocytes. (**B**) Representative images of histological sections at a higher magnification are shown (63X)
