# Supplementary material for: Drosophila EGFR pathway coordinates stem cell proliferation and gut remodeling following infection
Source: BMC Biol. 2010 Dec 22;8:152. doi: 10.1186/1741-7007-8-152 (PMC3022776; doi:10.1186/1741-7007-8-152)
Supplement: Additional file 4 — The EGFR pathway is required for enterocyte delamination upon infection. [file 1741-7007-8-152-S4.PDF]

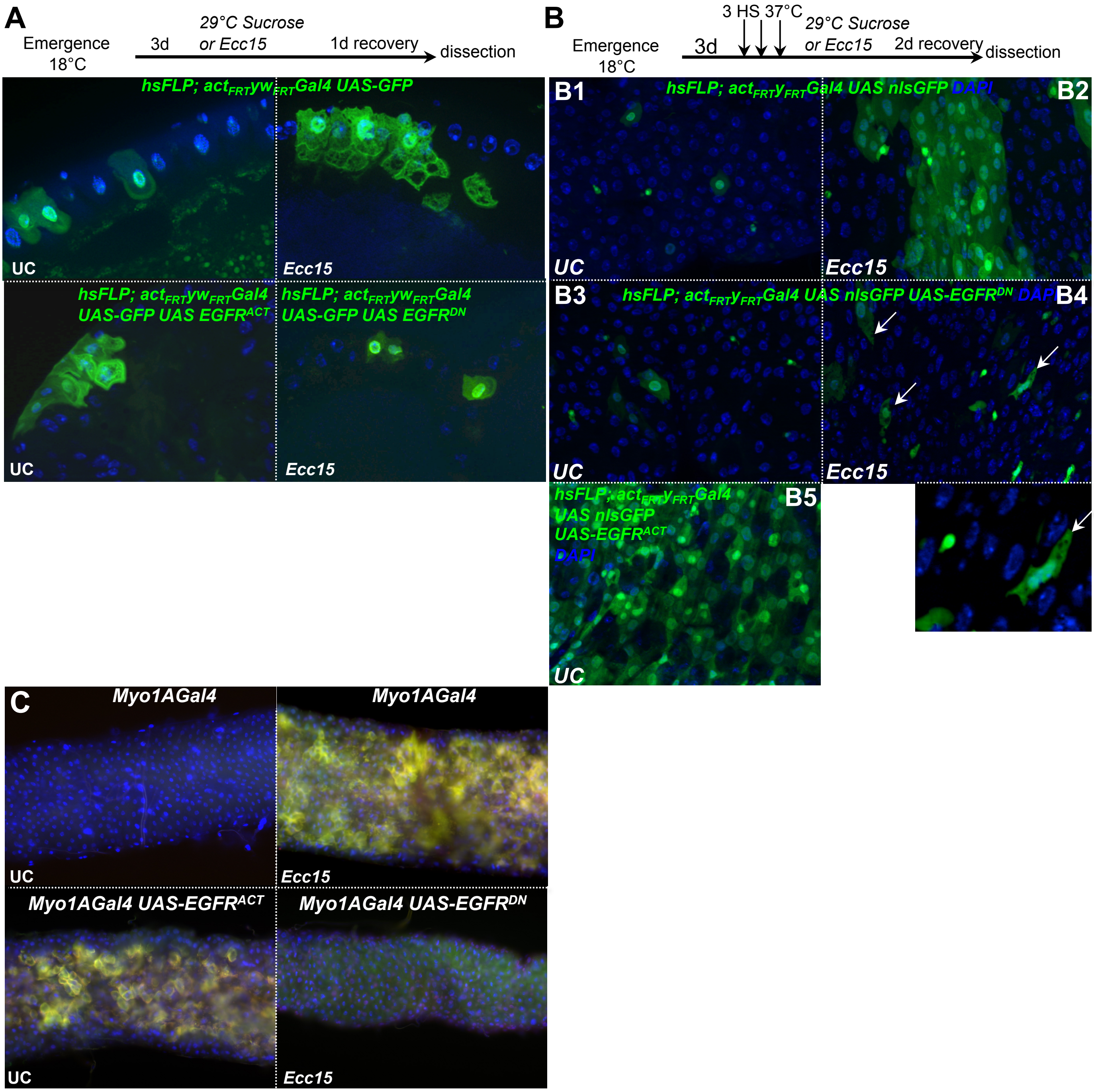

Additional file 4. The EGFR pathway is required for enterocyte delamination upon infection. (A) Enterocytes of wild-type flies randomly labeled with GFP by FLP-Out activation (29°C induction) of the *act-Gal4* driver reveals the delamination of cells in the gut of infected flies (see protocol above figure and methods for more details). In contrast, clones of GFP enterocytes deficient for EGFR (*UAS-EGFR^{DN}*) remain in the epithelium following infection. The activation of EGFR (*UAS-EGFR^{ACT}*) in enterocytes is sufficient to provoke delamination in the absence of infection. (**B**) The random induction of GFP-positive clones by heat-shocked-induced FLP-Out activation of the *act-Gal4* driver (see protocol above figure and methods for more details) in the midgut labels both precursor cells (small nuclei) and isolated enterocytes in unchallenged flies (**B1**). Upon infection, isolated enterocytes expressing GFP were not observed probably due to their delamination. Instead large patches of GFP labeled clones were observed that derived from the proliferation and differentiation of GFP precursors cells (**B2**). Clones of GFP cells (dispersed enterocytes and progenitors) expressing a dominant negative form of EGFR were observed in absence of infection (**B3**). Upon infection, no extension of clones deriving from GFP progenitors was observed, although single dispersed and highly vacuolized (white arrows and zoom) enterocytes were still observed after two days of infection (**B4**). This is consistent with the notion that EGFR is required in ISCs for stem cell proliferation as well as in enterocyte for their delamination. GFP clones expressing an activated form of EGFR are hyper-proliferative and rapidly colonize the whole gut tissue in absence of infection (**B5**). **(C)** Representative images were taken of wild-type guts, guts with enterocytes depleted of EGFR, or guts expressing an activated form of EGFR in enterocytes. Guts from 3-4 day old flies were stained with DAPI. Infection induces a characteristic yellow auto-fluorescence probably caused by the accumulation of lysing cells in the lumen. Guts of flies depleted for the EGFR pathway in enterocytes did not show this characteristic auto-fluorescence upon infection. Conversely, guts of flies expressing an activated form of EGFR in enterocytes displayed this auto-fluorescence in the absence of infection.
