# Supplementary material for: Drosophila EGFR pathway coordinates stem cell proliferation and gut remodeling following infection
Source: BMC Biol. 2010 Dec 22;8:152. doi: 10.1186/1741-7007-8-152 (PMC3022776; doi:10.1186/1741-7007-8-152)
Supplement: Additional file 5 — Enterocytes undergoing delamination display marks of increased autophagy. [file 1741-7007-8-152-S5.PDF]

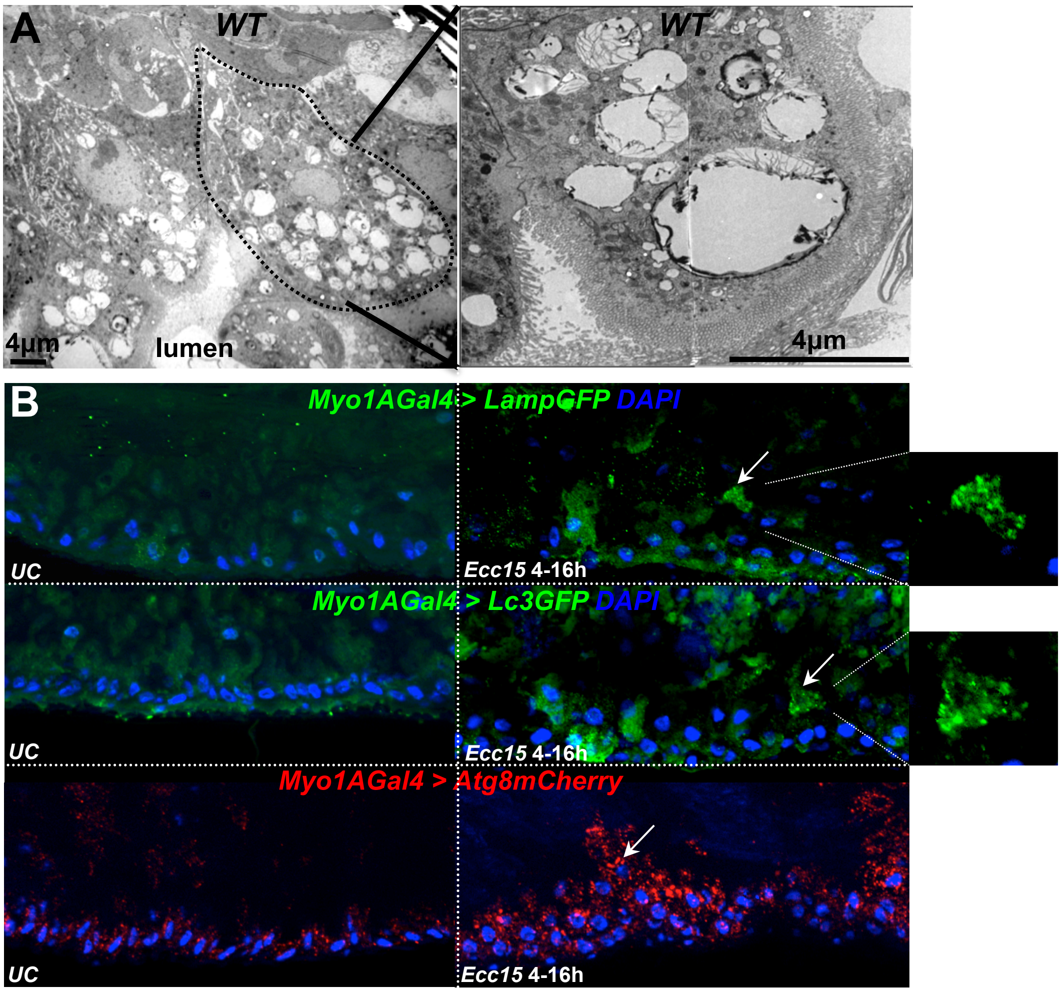

Additional file 5. Enterocytes undergoing delamination display marks of increased autophagy.

(A) Left. Representative image of electron microscopy scans of a wild-type infected gut showing that delaminating enterocytes (outlines with dotted line) undergo an intense vacuolization. Right. A higher magnification image showing vacuoles contain double membranes. (**B**) Infection with *Ecc15* induces the appearance of punctated signals positive for the autophagy markers Lamp, Lc3 and Atg8. Punctuated signals were mostly associated with delaminating cells (indicated with arrow in magnification).
