# Supplementary material for: Drosophila EGFR pathway coordinates stem cell proliferation and gut remodeling following infection
Source: BMC Biol. 2010 Dec 22;8:152. doi: 10.1186/1741-7007-8-152 (PMC3022776; doi:10.1186/1741-7007-8-152)
Supplement: Additional file 6 — The EGFR pathway is required in ISCs for the ISC proliferation induced by infection. [file 1741-7007-8-152-S6.PDF]

A

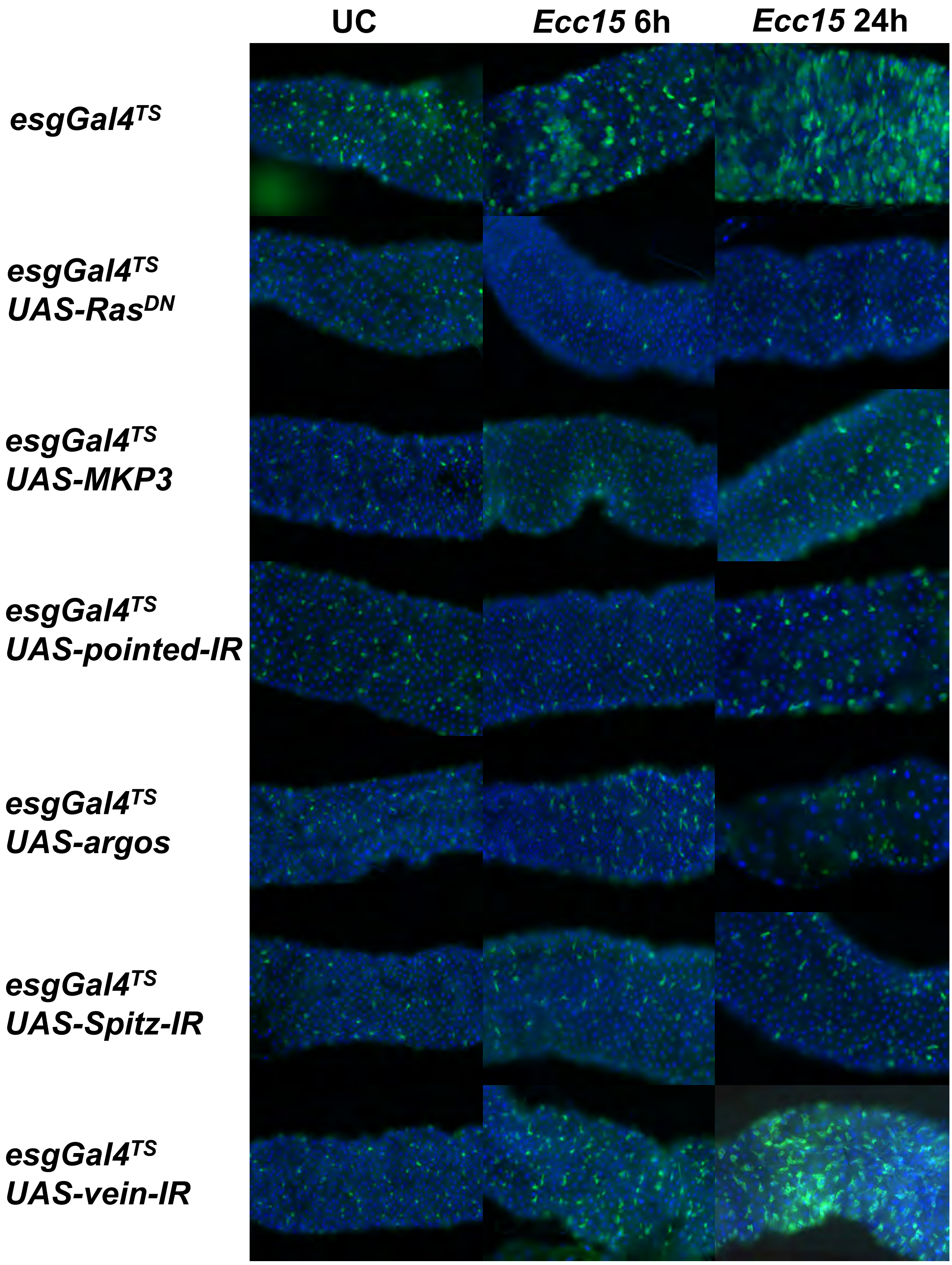
UC
Ecc15 6h
Ecc15 24h
esgGal4TS
esgGal4TS UAS-RasDN
esgGal4TS UAS-MKP3
esgGal4TS UAS-pointed-IR
esgGal4TS UAS-argos
esgGal4TS UAS-Spitz-IR
esgGal4TS UAS-vein-IR

B

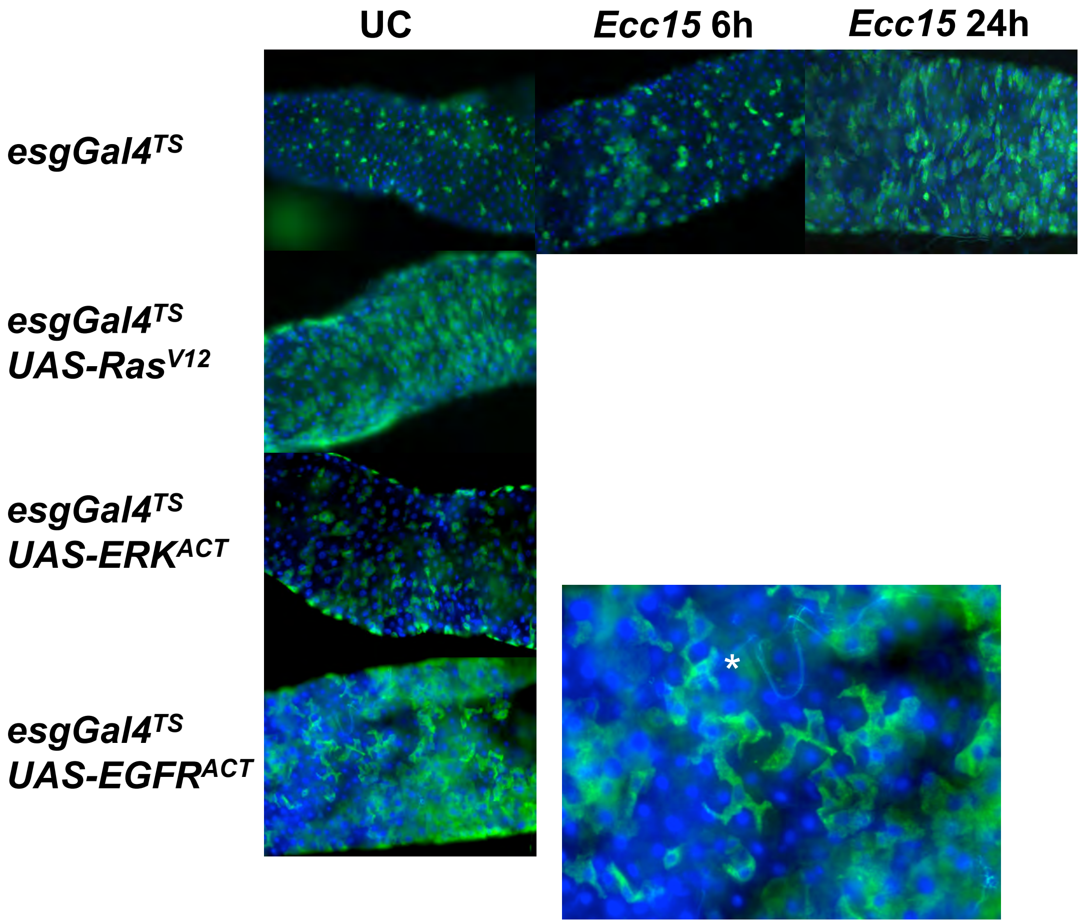

Additional file 6. The EGFR pathway is required in ISCs for the ISC proliferation induced by infection.
(A) Ingestion of *Ecc15* induced a marked increase in the number of *esgGal4^{TS} UAS-GFP*-positive cells (indicative of epithelium renewal), which was not observed when constructs inhibiting the EGFR pathway (*UAS-Ras^{DN}*, *UAS-MKP3, UAS-Pointed-IR, or UAS-argos*) were expressed in ISCs. Additionally, expression in ISCs of RNAi against the ligand *Spitz* (*UAS-Spitz-IR)*, which is expressed in ISCs, reduced the number of *esgGal4^{TS}*, *UAS-GFP*-positive cells. Epithelium renewal was induced to wild-type levels when RNAi against the ligand Vein (*UAS-vein-IR)* was expressed in ISCs. **(B)** Over-expression of activated forms of components of the EGFR pathway (*UAS-Ras^{V12}*, *UAS-EGFR^{ACT} or UAS-ERK^{ACT}* albeit to a much lesser extent*)* in ISCs was sufficient to induce a high level of epithelium renewal in the absence of infection. Interestingly, activation of EGFR pathway in ISCs generates a sub-population of cells with abnormal shape, which infiltrate the neighboring tissue (see * in the magnification).
