# Supplementary material for: Drosophila EGFR pathway coordinates stem cell proliferation and gut remodeling following infection
Source: BMC Biol. 2010 Dec 22;8:152. doi: 10.1186/1741-7007-8-152 (PMC3022776; doi:10.1186/1741-7007-8-152)
Supplement: Additional file 7 — Flies with a reduced level of EGFR pathway activity in ISCs display an increased susceptibility to Ecc15 infection. [file 1741-7007-8-152-S7.PDF]

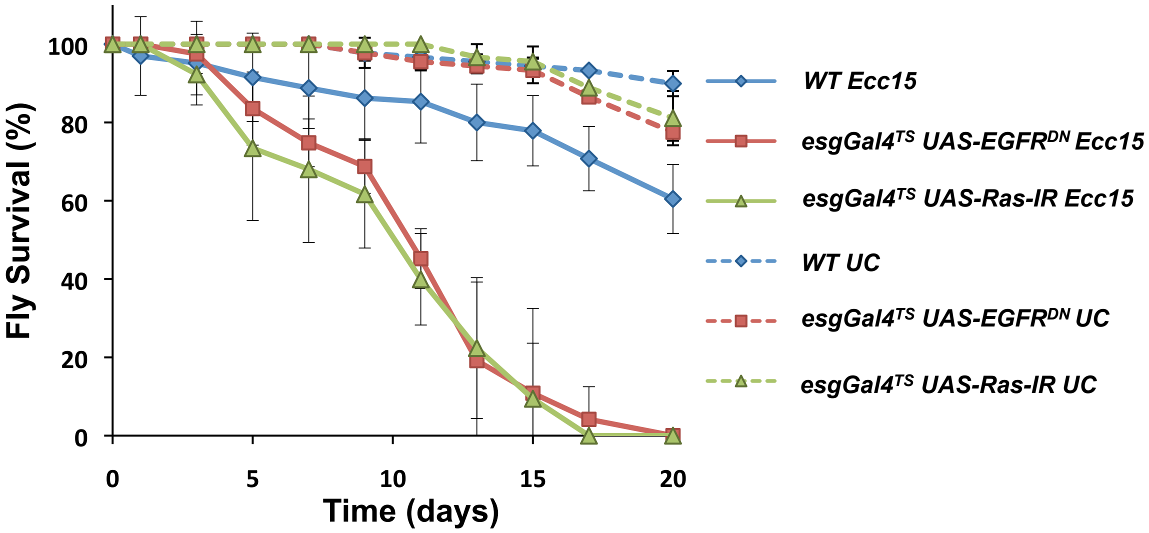

Additional file 7. Flies with a reduced level of EGFR pathway activity in ISCs display an increased susceptibility to *Ecc15* infection.

Survival analysis at 29°C shows that flies impaired in epithelium renewal due to a lack of EGFR pathway activity in ISCs succumbed 12–16 days following ingestion of *Ecc15*. The *UAS-EGFR*^{DN} and *UAS-Ras-IR* constructs were expressed in adult ISCs using *esgGal4*^{TS}.
