# Supplementary material for: Drosophila EGFR pathway coordinates stem cell proliferation and gut remodeling following infection
Source: BMC Biol. 2010 Dec 22;8:152. doi: 10.1186/1741-7007-8-152 (PMC3022776; doi:10.1186/1741-7007-8-152)
Supplement: Additional file 8 — The EGFR ligands Spitz and Keren are expressed in the progenitor cells. [file 1741-7007-8-152-S8.PDF]

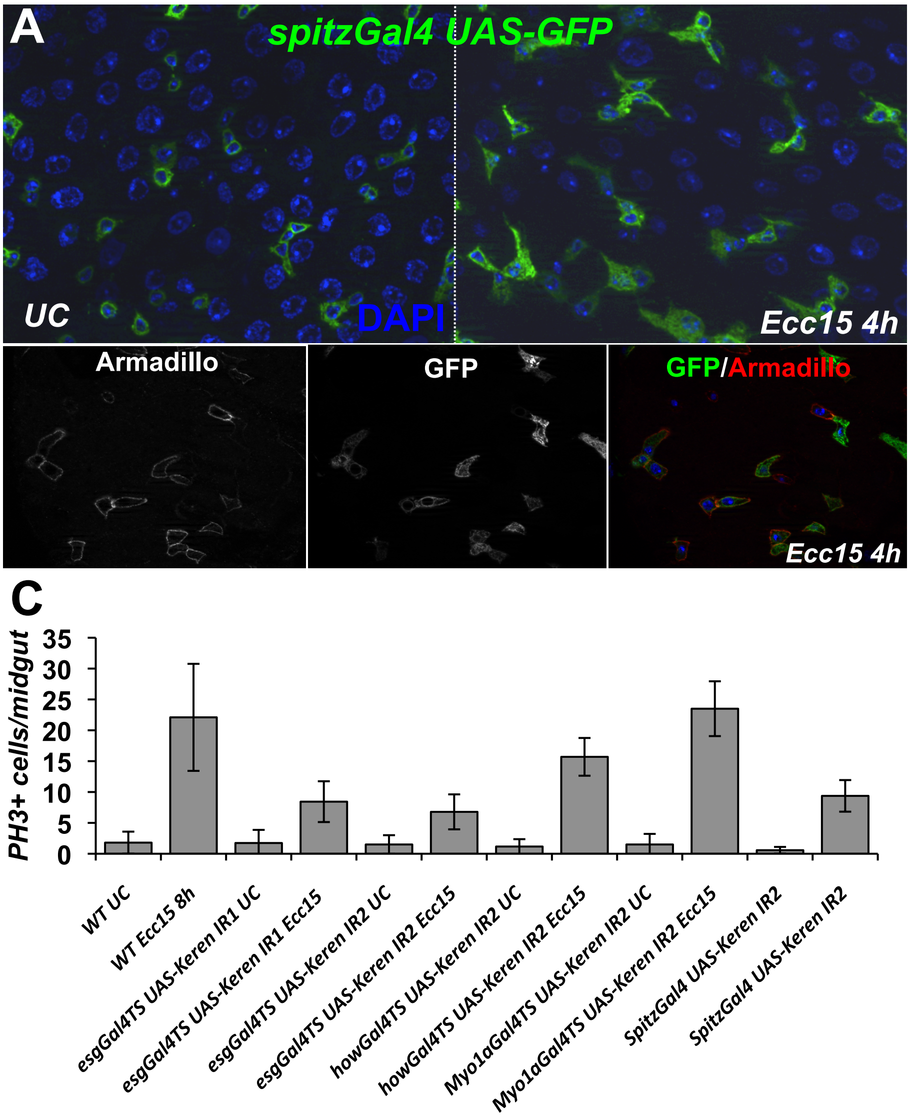

Additional file 8. The EGFR ligands Spitz and Keren are expressed in the progenitor cells.
(**A**) Use of *spitz-Gal4, UAS-GFP* flies revealed that *spitz* is expressed in progenitor cells both in unchallenged guts and guts infected with *Ecc15*. Co-staining with armadillo (red) indicated that both ISCs and enteroblasts are GFP positive. (**B)** Knock-down of *Keren* in precursor cells (using *esgGal4*^{TS}), but not in visceral muscles (using *howGal4*^{TS}) or enterocytes (using *Myo1AGal4*^{TS}) decreased the ISC proliferation induced by infection (shown as the number of PH3-positive cells per midgut). Two distinct RNAi lines targeting *Keren* expression were used (Additional file 16). Additionally, RNAi silencing of *Keren* expression using *spitzGal4* driver reduced the mitotic index supporting a requirement of Keren in progenitor cells.
