# Supplementary material for: Drosophila EGFR pathway coordinates stem cell proliferation and gut remodeling following infection
Source: BMC Biol. 2010 Dec 22;8:152. doi: 10.1186/1741-7007-8-152 (PMC3022776; doi:10.1186/1741-7007-8-152)
Supplement: Additional file 9 — The JAK/STAT pathway is activated in the visceral muscles. [file 1741-7007-8-152-S9.PDF]

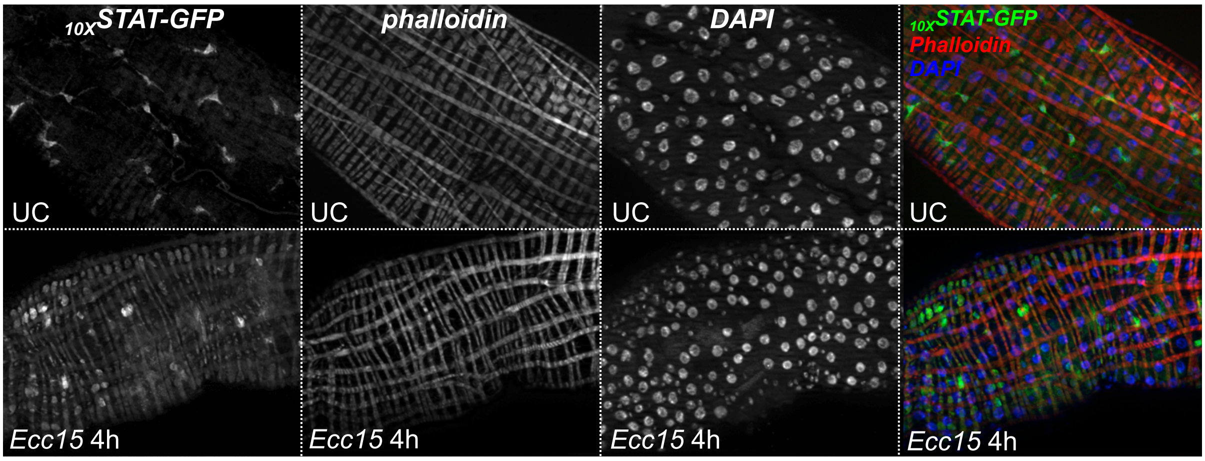

Additional file 9. JAK/STAT pathway is activated in the visceral muscles.
Apotome sections of the basal surface of the gut of $_{10X}$*STAT-GFP* flies were co-stained by rhodamine phalloidin (marking F-actin that is highly enriched in visceral muscles) and with an anti-GFP antibody (indicative of JAK/STAT pathway activity). In unchallenged conditions, the JAK/STAT pathway activity was found in precursor cells (upper panels) but not in visceral muscles (phalloidin staining). Upon infection with *Ecc15* the JAK/STAT pathway activity was detected in circular visceral muscles (marked with phalloidin) in addition to the previously reported activation in progenitor cells and enterocytes (see text and Figure 4).
