# Supplementary material for: Drosophila EGFR pathway coordinates stem cell proliferation and gut remodeling following infection
Source: BMC Biol. 2010 Dec 22;8:152. doi: 10.1186/1741-7007-8-152 (PMC3022776; doi:10.1186/1741-7007-8-152)
Supplement: Additional file 10 — EGFR and JAK/STAT pathways synergize in ISCs to promote proliferation. [file 1741-7007-8-152-S10.PDF]

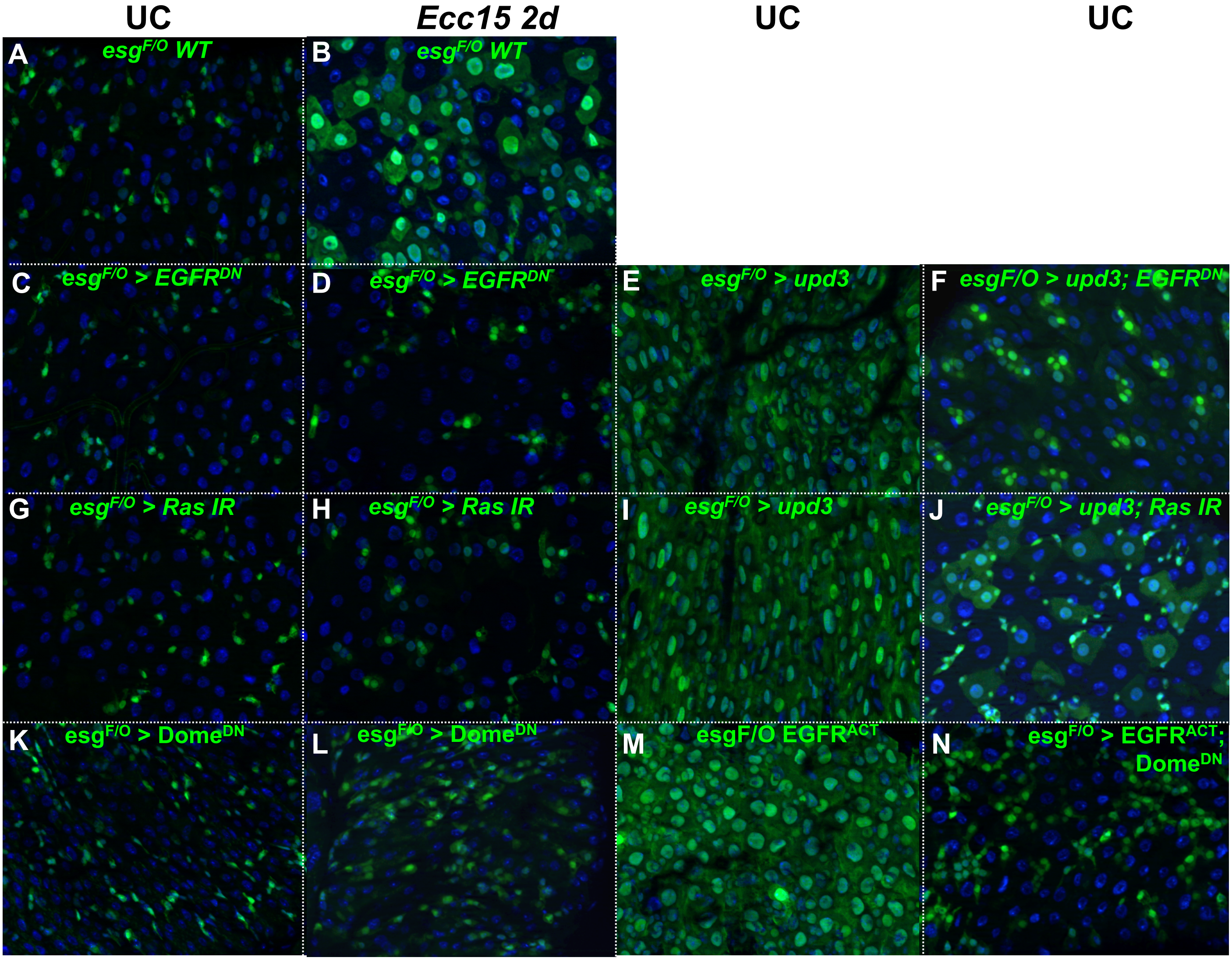

Additional file 10. EGFR and JAK/STAT pathways synergize in ISCs to promote proliferation. Epistasis analysis using the *esg-Flip*-Out system (*esg*$^{F/O,}$, [10]). Flies (*esgGal4*^{TS} *UAS-FLP tub*$_{FRT}$*CD2*$_{FRT}$*Gal4 UAS-GFP*; *UAS-transgene*) were raised at 18°C during their development and switched to 29°C at 3 days of age. This results in the excision of a yellow/stop cassette and the subsequent activation of the *actGal4* driver in all progenitor cells. Progenitor cells and their progeny subsequently express the indicated transgene and are labeled by GFP. The expansion of the GFP labeled compartment is a direct read-out of the rate of epithelium renewal. As reported previously, infection induces a strong increase in the rate of epithelium renewal (**A**, **B**). This increase is not observed when ISCs expressed constructs that reduced EGFR (*UAS-EGFR*^{DN} and *UAS-Ras IR,* **D**, **H**) or JAK/STAT (*UAS-Dome*^{DN}*,* **L**) signaling. In absence of infection, expression of Upd3 was sufficient to induce high levels of epithelium renewal (**E, I**) that were suppressed upon co-expression of inhibitors of the EGFR pathway (*UAS-EGFR*^{DN} and *UAS-Ras IR,* **F, J**). Expression of an activated form of EGFR was sufficient to induce high levels of epithelium renewal (**M**) that was suppressed upon co-expression of a dominant negative form of Domeless (**N**). Of note, activation of the EGFR pathway did not rescue the lack of differentiation observed in JAK/STAT deficient clones (**N**). Collectively, these results indicate that both the JAK/STAT and EGFR pathways are required for ISC proliferation, but that the JAK/STAT pathway has a unique role to promote progenitor differentiation.
