# Supplementary material for: Drosophila EGFR pathway coordinates stem cell proliferation and gut remodeling following infection
Source: BMC Biol. 2010 Dec 22;8:152. doi: 10.1186/1741-7007-8-152 (PMC3022776; doi:10.1186/1741-7007-8-152)
Supplement: Additional file 11 — Alteration of the EGFR pathway in enterocytes affects gut morphology. [file 1741-7007-8-152-S11.PDF]

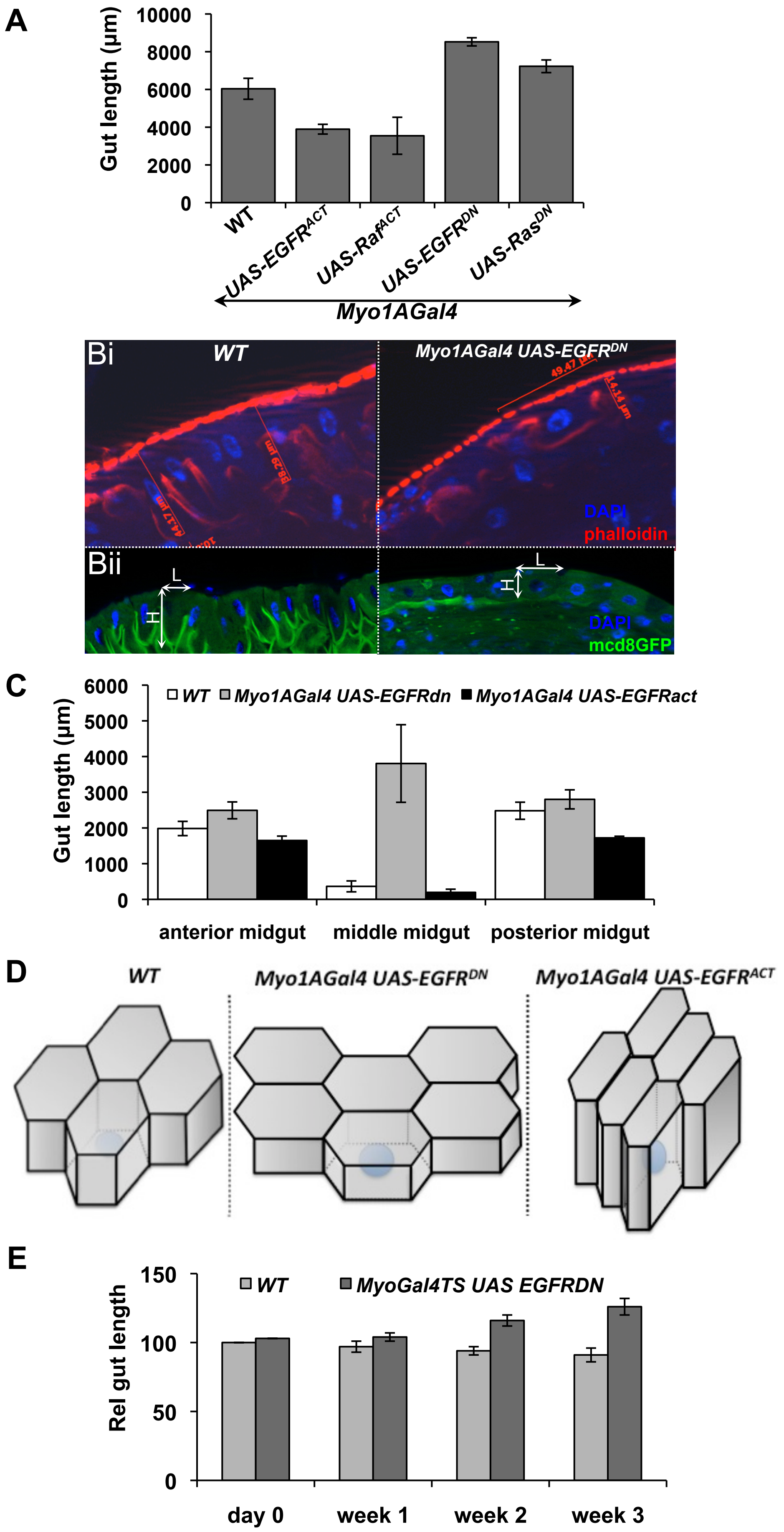
A
Gut length (µm)
10000
8000
6000
4000
2000
0
WT
UAS-EGFRACT
UAS-RafACT
UAS-EGFRDN
UAS-RasDN
Myo1AGal4
Bi
WT
Myo1AGal4 UAS-EGFRDN
DAPI
phalloidin
Bii
L
H
DAPI
mcd8GFP
C
Gut length (µm)
6000
5000
4000
3000
2000
1000
0
WT
Myo1AGal4 UAS-EGFRdn
Myo1AGal4 UAS-EGFRact
anterior midgut
middle midgut
posterior midgut
D
WT
Myo1AGal4 UAS-EGFRDN
Myo1AGal4 UAS-EGFRACT
E
Rel gut length
150
100
50
0
WT
MyoGal4TS UAS EGFRDN
day 0
week 1
week 2
week 3

Additional file 11. Alteration of the EGFR pathway in enterocytes affects gut morphology.
(A) Expression of dominant negative forms of components of the EGFR pathway (*UAS-EGFR^{DN}, UAS-Ras^{DN}*) increased midgut length, whereas over-expression of active forms of components of the EGFR pathway (*UAS-EGFR^{ACT}, UAS-Raf^{ACT})* reduced gut length. Constructs were expressed in enterocytes using the *Myo1AGal4* driver. **(B)** Phalloidin staining (Bi) or live imaging of flies expressing *mcd8-GFP* in enterocytes (Bii) reveal the flattening of enterocytes expressing a dominant negative form of EGFR. Transversal optic sections of guts derived from 4 day-old flies and stained with DAPI and phalloidin (Bi) or DAPI alone (Bii). L=Length; H=Height **(C)** The increase in gut length was largely due to an elongation of enterocytes in a middle region of the midgut (around the copper cells region), where the epithelium is composed of columnar cells and is folded within the fly abdomen. **(D)** Schematic representation of the effect of the role of the EGFR pathway on enterocyte shape. **(E)** The relative length of guts from flies with wild-type enterocytes and EGFR depleted enterocytes (*Myo1AGal4^{TS}, UAS-EGFR^{DN})* at 3, 7, 14 and 21 days of age are shown. Flies where shifted to 29°C at 3 days post eclosion.
