# Supplementary material for: Drosophila EGFR pathway coordinates stem cell proliferation and gut remodeling following infection
Source: BMC Biol. 2010 Dec 22;8:152. doi: 10.1186/1741-7007-8-152 (PMC3022776; doi:10.1186/1741-7007-8-152)
Supplement: Additional file 12 — Flies with reduced EGFR activity in enterocytes are highly susceptible to oral infection with Ecc15. [file 1741-7007-8-152-S12.PDF]

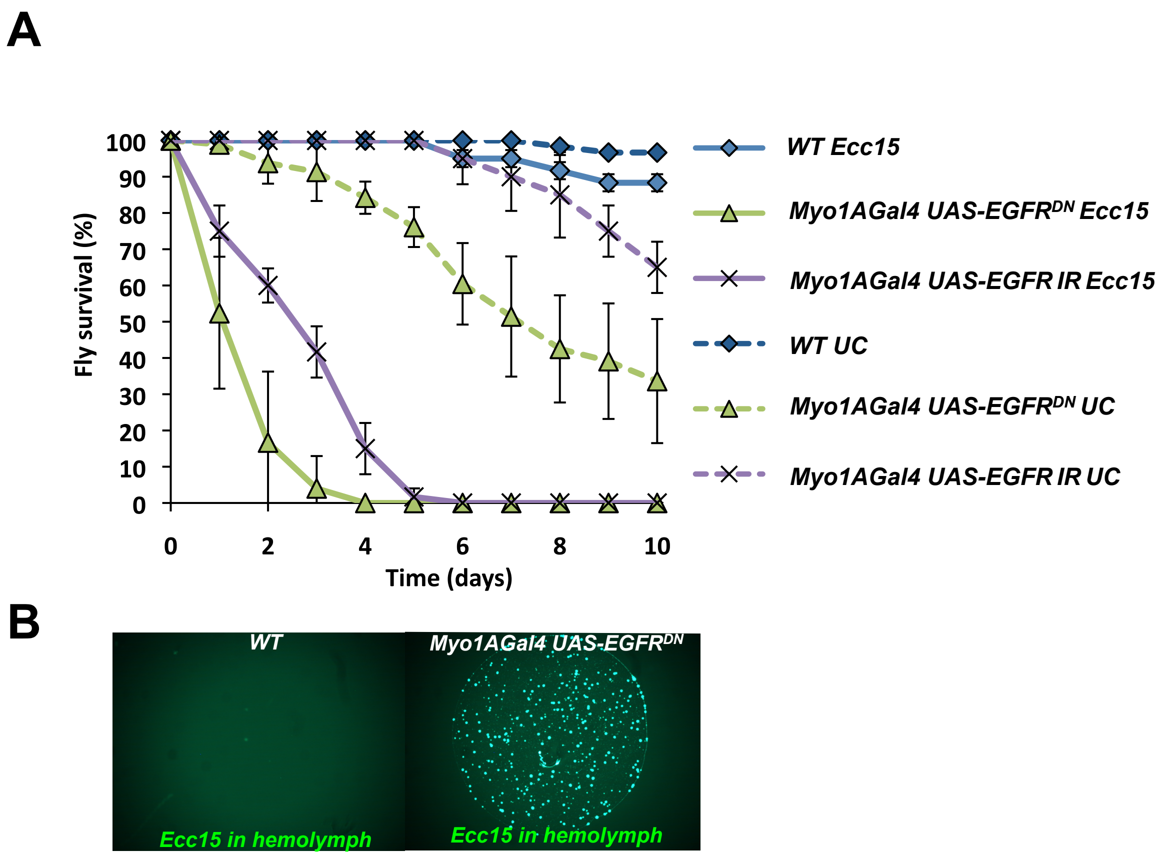

Additional file 12. Flies with reduced EGFR activity in enterocytes are highly susceptible to oral infection with *Ecc15*.

(**A**) Survival curves of wild-type flies or flies with enterocytes depleted of EGFR activity (*UAS-EGFR^{DN}, UAS-EGFR-IR*), following ingestion of *Ecc15* or sucrose (unchallenged (UC) control) are shown. **(B)** Representative plates of *Ecc15-GFP* (CFUs/fly) recovered from hemolymph of flies with wild-type enterocytes or enterocytes depleted of the EGFR (*UAS-EGFR^{DN})* and observed by fluorescence microscopy. In contrast to wild-type flies, *Ecc15-GFP* bacteria were recovered from flies with reduced EGFR activity in enterocytes.
