# Supplementary material for: Drosophila EGFR pathway coordinates stem cell proliferation and gut remodeling following infection
Source: BMC Biol. 2010 Dec 22;8:152. doi: 10.1186/1741-7007-8-152 (PMC3022776; doi:10.1186/1741-7007-8-152)
Supplement: Additional file 13 — Ecc15 infection alters adherens junction dynamics in the gut. [file 1741-7007-8-152-S13.PDF]

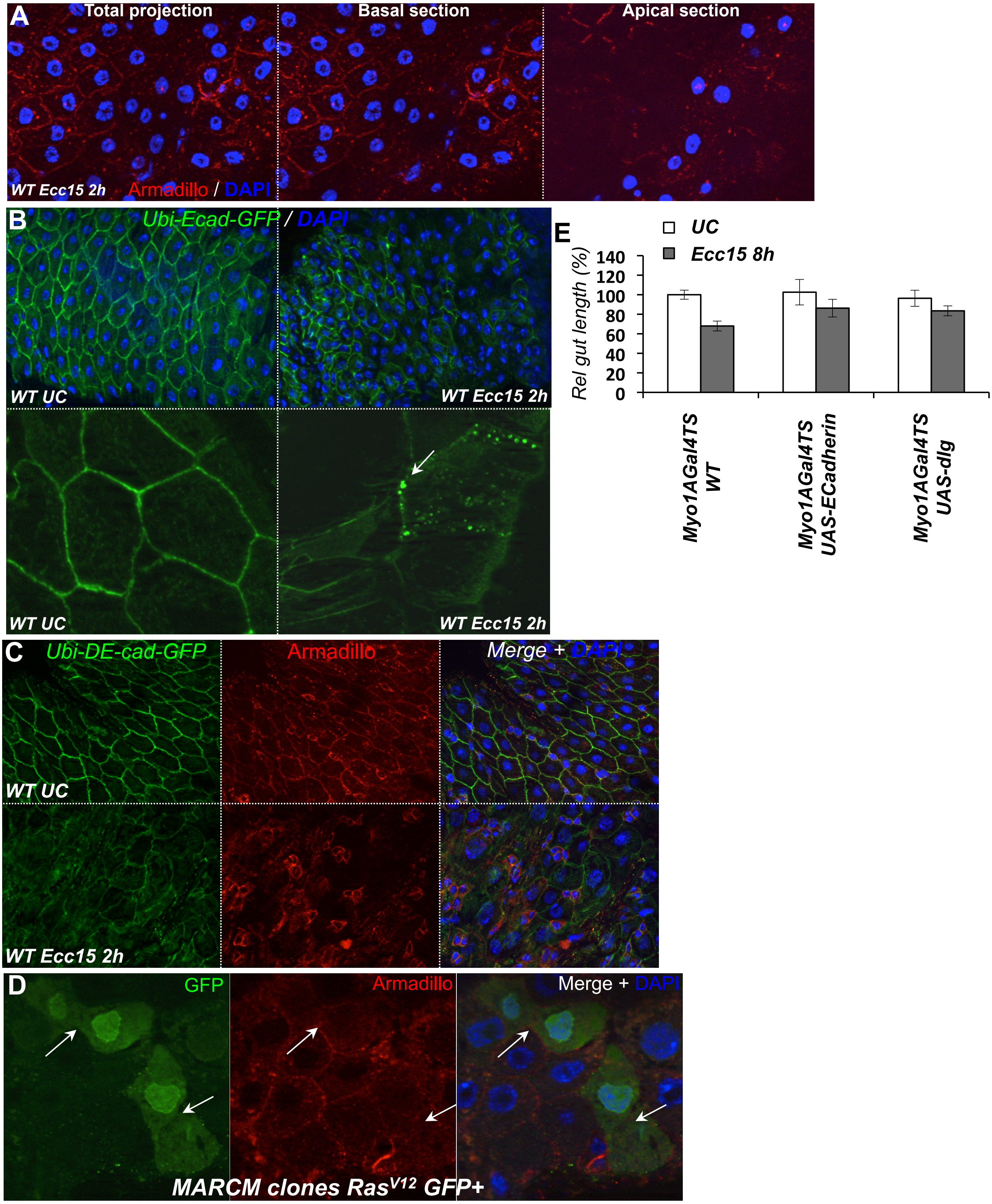
A
Total projection
Basal section
Apical section
WT Ecc15 2h
Armadillo / DAPI
B
Ubi-Ecad-GFP / DAPI
WT UC
WT Ecc15 2h
WT UC
WT Ecc15 2h
E
UC
Ecc15 8h
Rel gut length (%)
140
120
100
80
60
40
20
0
Myo1AGal4TS WT
Myo1AGal4TS UAS-ECadherin
Myo1AGal4TS UAS-dlg
C
Ubi-DE-cad-GFP
Armadillo
Merge + DAPI
WT UC
WT Ecc15 2h
D
GFP
Armadillo
Merge + DAPI
MARCM clones Ras^{V12} GFP+

Additional file 13. *Ecc15* infection alters adherens junction dynamics in the gut.

(A) Representative Z-stack sections of guts dissected 2 h post-infection and stained with an anti-Armadillo antibody and DAPI reveals that the loss of Armadillo upon infection occurs mainly in the most apical cells within the epithelium (luminal side). **(B)** Expression of an *ubi-DE-cadherin-GFP* in the gut of wild-type flies. The pattern of GFP expression is homogenous in uninfected guts and localized to the membrane of enterocytes. Upon infection, the pattern of GFP expression is disrupted and mostly relocalized to vesicles (arrow). **(C)** Co-staining of Armadillo (red) and DE-cadherin (green) in guts of unchallenged and *Ecc15* infected flies (t=2 h) revealed similar dynamics upon infection. **(D)** Enterocytes of flies randomly labeled with GFP and expressing the Ras^{V12} (*Ras* constitutively active) oncogene by FRT/FLP activation in polyploid cells reveals that Armadillo staining (red) is disrupted at the junction (arrows) between these cells (green, GFP). **(E)** Overexpression of the adherens junction component E-Cadherin (*UAS-E-Cadherin*) or the septate junction component Discs large (*UAS-dlg*) in enterocytes reduces the shortening of the gut that normally occurs upon infection.
