# Supplementary material for: Drosophila EGFR pathway coordinates stem cell proliferation and gut remodeling following infection
Source: BMC Biol. 2010 Dec 22;8:152. doi: 10.1186/1741-7007-8-152 (PMC3022776; doi:10.1186/1741-7007-8-152)
Supplement: Additional file 14 — ISCs are in close proximity to visceral muscles. [file 1741-7007-8-152-S14.PDF]

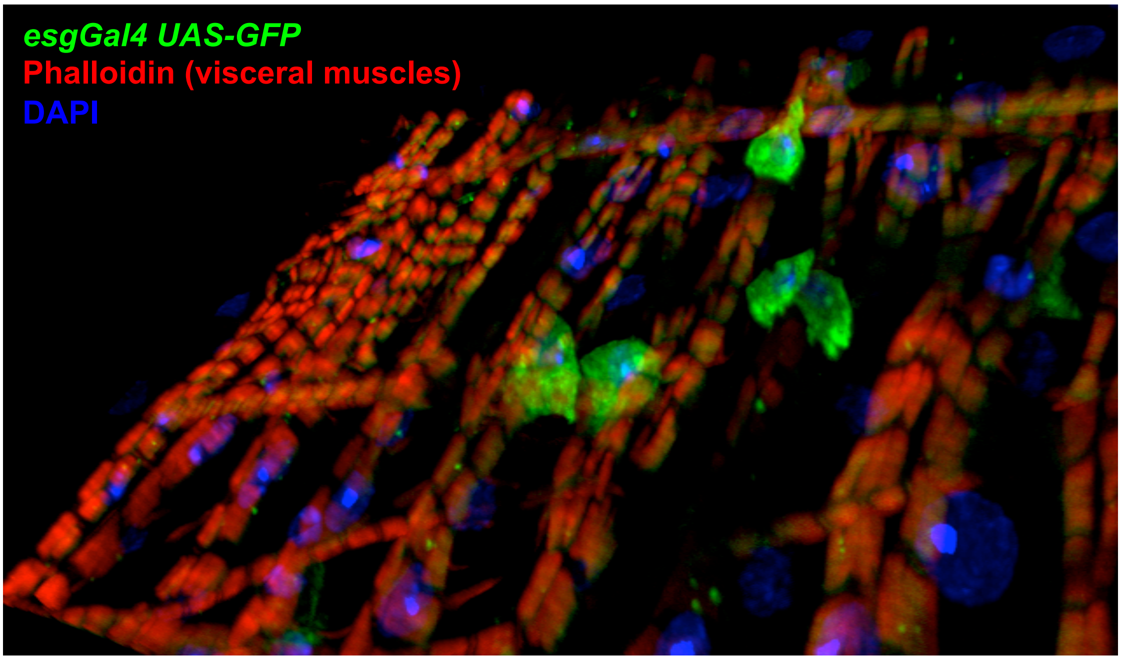

Additional file 14. ISCs are in close proximity to visceral muscles.
A 3-D reconstruction from a Z-stack image of the *Drosophila* midgut reveals the proximity of ISCs (green, *esgGal4^{TS} UAS-GFP*) and visceral muscle (red, anti-phalloidin staining). The localization of enterocytes (blue, large nuclei as revealed by DAPI) is more apical than ISCs.
