# Supplementary material for: Drosophila EGFR pathway coordinates stem cell proliferation and gut remodeling following infection
Source: BMC Biol. 2010 Dec 22;8:152. doi: 10.1186/1741-7007-8-152 (PMC3022776; doi:10.1186/1741-7007-8-152)
Supplement: Additional file 15 — The EGFR pathway is required in both enteroblasts and enterocytes for proper morphogenesis. [file 1741-7007-8-152-S15.PDF]

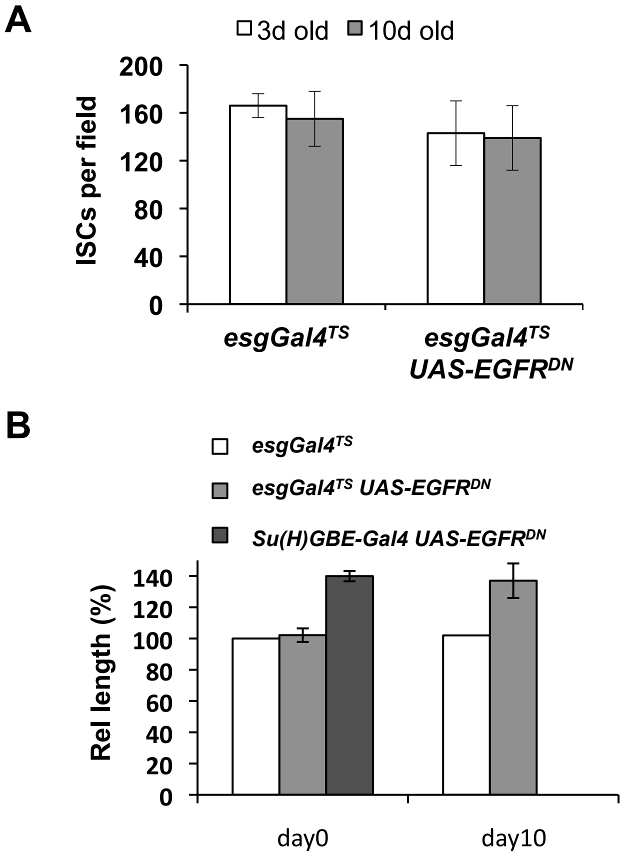

Additional file 15. The EGFR pathway is required in both enteroblasts and enterocytes for proper morphogenesis.

(A) The EGFR pathway is not required for ISC maintenance. The effect of EGFR on ISC maintenance was determined by comparing the number of GFP-positive cells in the guts of 3 and 10 day-old flies with either wild-type ISCs (*esgGal4^{TS} UAS-GFP)* or EGFR-depleted ISCs (*esgGal4^{TS} UAS-EGFR^{DN})*. Mean numbers of ISCs per midgut ± SE are shown. **(B)** EGFR modulates gut morphogenesis in both enteroblasts and enterocytes. The relative length of guts from flies with wild-type ISCs (*esgGal4^{TS} UAS-GFP)*, EGFR-depleted ISCs (*esgGal4^{TS}* UAS-*EGFR^{DN})* or EGFR-depleted enteroblasts (*Su(H)GBE-Gal4 UAS-EGFR^{DN}*) at 3 and 10 days of age are shown. Ten-day old flies expressing *Su(H)GBE-Gal4 UAS-EGFR^{DN}* could not be obtained for comparison.
