# Supplementary material for: Drosophila EGFR pathway coordinates stem cell proliferation and gut remodeling following infection
Source: BMC Biol. 2010 Dec 22;8:152. doi: 10.1186/1741-7007-8-152 (PMC3022776; doi:10.1186/1741-7007-8-152)
Supplement: Additional file 16 — List of fly stocks used in this study. [file 1741-7007-8-152-S16.PDF]

Drosophila stocks used in the study

stock name	genotype	donator	Reference
UAS-EGFR^{DN}	w;UAS-EGFRDN;UAS-EGFRDN	Bloomington	Ninov et al.2009
UAS-Ras^{DN}	w,UASRas85D^{N17}	Bloomington	Jiang et al. 2009a
UAS-Raf^{DN}	w;LF/CyO;UAS-Raf^{DN}/TM2	JC Pastor-Pareja	Jiang et al. 2009a
UAS-EGFR-IR	w;UAS-EGFR-IR	VDRC	-
UAS-Ras-IR	w;;UAS-Ras-IR3	NIG	-
UAS-Raf-IR	w;UAS-Raf-IR1	NIG	-
UAS-EGFR^{ACT}	w;;UAS-TorD-DER	M Freeman	Brown et al. 2006
UAS-Ras^{V12}	w;UAS-Ras^{V12}; sb/TM6B	JC Pastor-Pareja	Wu et al. 2010
UAS-Raf^{GOF}	w;;UAS-phl^{GOF}	JC Pastor-Pareja	-
UAS-ERK^{ACT}	yw;;UAS-ERK^{ACT}	JC Pastor-Pareja	-
vein-nls-LacZ	yw;vn-lacZ(P1719),FRT82B/TM6B	BA Edgar	Jiang et al. 2009a
UAS-vein1.2	w;;UAS-vein$^{1.2}$	BA Edgar	Jiang et al. 2009a
UAS-vein-IR	w; UAS-vein-IR	VDRC	Jiang et al. 2009a
UAS-sKeren	w;;UASsecr-Keren	D Montell	McDonald et al. 2006
UAS-Keren-IR1	w;UAS-mKeren-IR	D Montell	McDonald et al. 2006
UAS-Keren-IR2	w; UAS-Keren IR	NIG	-
SptizGal4	SpiGal4$^{(NP0261)}$	NIG	Jiang et al. 2009a
UAS-sSpitz	w;UAS-sSPI	D Montell	McDonald et al. 2006
UAS-Spitz-IR	w;UAS-Spitz-IR	VDRC	-
UAS-MKP3	yw;sp/CyO;UAS-MKP3/TM6B	BA Edgar	Jiang et al. 2009a
UAS-argos	w; UAS-argos^{232}	JC Pastor-Pareja	-
STAT-GFP	w;10XSTAT-GFP	M Crozatier	Bach et al. 2007
esgGal4^{TS}	w;esgGal4, Gal80TS	C Micchelli	Beebe et al. 2009
HowGal4^{TS}	w;Gal80TS/CyO;howGal4^{24B}	BA Edgar	Jiang et al. 2009a
Myo1AGal4^{TS}	w;Myo1AGal4;Gal80TS	BA Edgar	Jiang et al. 2009b
UAS-Upd3	w;UAS-Upd3/CyO	M Meister	Buchon et al. 2009
UAS-Upd3-IR	w;UAS-Upd3-IR	M Crozatier	Agaisse et al. 2003
UAS-Dome	w;;UAS-PDomeGFP	M Crozatier	Krzemien et al.2007
UAS-Dome^{DN}	w;;UAS-Dome^{DN}	M Crozatier	Brown et al.2001
UAS-STAT-IR	w;;UAS-STAT-IR1/TM3	NIG	Buchon et al. 2009
UAS-Socs36E	w;UAS-Socs36E	M Crozatier	Krzemien et al.2007
Ubi-Ecad-GFP	w;Ubi-DE-cad-GFP	JC Pastor-Pareja	Maeda et al. 2008
clonal system for delamination	yw,hsFLP; actFRTywFRTGal4,UASGFP/CyO	JC Pastor-Pareja	Wu et al. 2010
MARCM Rasv12	yw,hsFLP;act>>Gal4;UAS-GFP/CyO; Gal80,FRT82B	W Deng	Poulton et al. 2006
MARCM Rasv12	w;UAS-RasV12; FRT82B	W Deng	Poulton et al. 2006
puc-LacZ	puc^{E69}	H Agaisse	Agaisse et al. 2003
UAS-LampGFP	w; UAS-Lamp-GFP	T Neufeld	Arsham et al. 2009
UAS-Lc3GFP	w; UAS-huLc3GFP	Bloomington	-
UAS-Atg8mCherry	w; UAS-mCherry-Atg8a	T Neufeld	Arsham et al. 2009
esgF/O	esgGal4^{TS} UAS-FLP tub$_{FRT}$CD2$_{FRT}$Gal4 UAS-GFP	BA Edgar	Jiang et al. 2009b
Su(H)GbeGal4	w;; Su(H)GbeGal4/TM3sb	SX Hou	Zeng et al. 2010
deltaGal4	w;; deltaGal4/TM6Tb	SX Hou	Zeng et al. 2010
dlgGFP	w,dlg-GFP	FlyTrap	Kelso et al. 2004
UAS-dlg	UAS-dlgYFP	D StJohnston	-
UAS-Ecad	UAS-ECadherin-GFP	JC Pastor-Pareja	-

Additional file 16. List of fly stocks used in this study.
